# Dysregulated Sp1/miR-130b-3p/HOXA5 axis contributes to tumor angiogenesis and progression of hepatocellular carcinoma

**DOI:** 10.7150/thno.43640

**Published:** 2020-04-06

**Authors:** Yadi Liao, Chenwei Wang, Zhiwen Yang, Wenwu Liu, Yichuan Yuan, Kai Li, Yuanping Zhang, Yongjin Wang, Yunxing Shi, Yuxiong Qiu, Dinglan Zuo, Wei He, Jiliang Qiu, Xinyuan Guan, Yunfei Yuan, Binkui Li

**Affiliations:** 1 State Key Laboratory of Oncology in South China, Collaborative Innovation Center for Cancer Medicine, Sun Yat-Sen University Cancer Center, Guangzhou 510060, P. R. China; 2 Department of Liver Surgery, Sun Yat-Sen University Cancer Center, Guangzhou 510060, P. R. China; 3 Department of Clinical Oncology, The University of Hong Kong, 852 Hong Kong, P. R. China

**Keywords:** Hepatocellular carcinoma, Angiogenesis, miR-130b-3p, HOXA5

## Abstract

Angiogenesis, one of the hallmarks of cancer, is essential for both tumor growth and metastasis. However, its molecular mechanisms in hepatocellular carcinoma (HCC) are largely unknown. Here, we report the role of HOXA5 in tumor angiogenesis of HCC.

**Methods**: The expression of miR-130b-3p and HOXA5 was determined by qRT-PCR and immunohistochemistry, respectively. Capillary tube formation assay, chicken chorioallantoic membrane assay, and subcutaneous xenograft experiments were performed to investigate the role of miR-130-3p and HOXA5. Luciferase reporter assay and chromatin immunoprecipitation assay were performed to evaluate the interaction between Sp1, miR-130b-3p and HOXA5.

**Results**: miR-130b-3p was found up-regulated in HCC and correlated with a poor prognosis. miR-130b-3p promoted HCC angiogenesis both *in vitro* and *in vivo*. Mechanistically, HOXA5 was validated as a direct target of miR-130b-3p. Furthermore, we demonstrated that HOXA5 was down-regulated in HCC and its down-regulation was associated with larger tumor size, shorter overall survival, and higher recurrence probability. Moreover, HOXA5 was significantly associated with angiogenesis biomarkers such as CD31 and CD34. Functional studies revealed that the knockdown of HOXA5 also significantly promoted HCC angiogenesis both *in vitro* and *in vivo*. Knocking-down HOXA5 significantly provoked HCC cells to induce the capillary tube formation, migration and proliferation of endothelial cells. In xenograft animal models, we found that a decrease of HOXA5 effectively enhanced tumor growth and increased microvessel densities. We further demonstrated that miR-130b-3p could be directly transcriptionally regulated by Sp1.

**Conclusions**: This study showed that a dysregulation in the Sp1/miR-130b-3p/HOXA5 axis contributed to HCC progression and angiogenesis, and that HOXA5 can be considered as a promising therapeutic target for treating HCC.

## Introduction

Angiogenesis is recognized as one of the hallmarks of cancer and essential for both tumor growth and metastasis 1, 2. Hepatocellular carcinoma (HCC) is a hypervascular tumor with frequent intrahepatic and extrahepatic metastasis, which is responsible for the high recurrence rate and poor prognosis of HCC 3-5. Therefore, anti-angiogenesis has been considered as an attractive therapeutic strategy for HCC.

The involvement of proangiogenic molecules, such as VEGF, VEGF-C/VEGFR3, Ang2/Tie2, in cancer development have been previously described 6. However, the molecular mechanisms of tumor angiogenesis in HCC is yet to be fully elucidated. Currently, targeted agents including sorafenib, regofenib and lenvatinib, are being used for HCC treatment 7. Of the multiple effects on tumors, the anti-angiogenic effect is fundamental to the clinical benefits of targeted therapies. Moreover, the combination of anti-angiogenic agents and immunotherapies could significantly improve tumor response rates 8. Thus, anti-angiogenic treatment is indispensable for HCC patients. The molecular mechanisms of HCC angiogenesis are under urgent need to be investigated.

MicroRNAs (miRNAs) are evolutionarily conserved small non-coding RNAs and are involved in nearly every biological process by targeting mRNAs for cleavage or translational repression. Emerging evidence indicates that the dysregulation of miRNA is directly implicated in the process of angiogenesis. For example, miR-93, miR-135b, and miR-205 exert pro-angiogenesis effects through the suppression of integrin-β8 9, HIF-1 10, and PTEN 11, respectively. On the other hand, miR-145, miR-15a/16, and miR-4306 exert an anti-angiogenesis activity by suppressing the expression of p70S6K1 12, VEGFA 13, and SIX1/Cdc42/VEGFA 14, respectively. Our previous study revealed that miR-130b-3p was up-regulated in HCC 15, however, its role in the regulation of tumor angiogenesis for HCC is still unclear.

HOXA5, a member of the HOX family, contains a conserved DNA binding domain, known as the homeobox-containing domain. The HOX family contains 39 HOX genes that are classified into four clusters namely A to D 16-18. Transcription factors encoded by the homeobox genes have a pivotal role in governing the process of tumor angiogenesis. Previous studies have indicated that HOXB9 promotes tumor cell proliferation and angiogenesis in breast cancer and is associated with poor clinical outcomes 19, 20. Overexpression of HOXC10 promotes angiogenesis in human glioma by interacting with PRMT5 21. The overexpression of HOXB13 was shown to be correlated with angiogenesis and poor prognosis in pancreatic carcinoma 22. However, the function of HOXA5 in HCC is still unclarified.

In this study, we demonstrated that HOXA5 was an HCC tumor suppressor. Its dysregulation was critical to enhance the angiogenic phenotype of HCC. Furthermore, HOXA5 was identified as an important prognostic predictor and could be a potential therapeutic target for treating HCC.

## Methods

### Human tissue specimens

All patients included in the present study underwent an initial surgical treatment at the Sun Yat-sen University Cancer Center (SYSUCC; Guangzhou, China), none of them received any local or systemic anticancer therapies before surgical resection, and no postoperative anticancer treatments were administered before any identified relapse. The diagnosis of all cases was confirmed pathologically based on the terminology criteria established by the International Working Party.

The studied patients were classified into 2 cohorts. Cohort 1 comprised of archived paraffin-embedded pathological specimens from 107 consecutive HCC patients who received treatment between July 2003 and July 2007. Cohort 2 comprised of a separate set of 449 HCC patients who were treated between December 2003 and May 2015. All patients' data were marked as anonymous prior to analysis.

Clinicalpathological characteristics are presented in Table S1 and Table S3, respectively. Written informed consent was obtained from each patient. The study was approved by the Institute Research Ethics Committee at the Cancer Center.

### Preparation of tumor cell-conditioned medium (TCM)

Stably-transfected tumor cells (3×10^6^) were seeded in a 10 cm dish and incubated for 48 h, after which the medium was removed and the cells were washed thrice with 1 × phosphate -buffered saline (PBS). All cells were then cultured in 10 mL serum-free medium (SFM) for 24 h. Following the incubation period, the TCM was collected and centrifuged at 3000 rpm (4 °C, 5 min) to remove detached cells and was then filtered through a 0.45 μm membrane (Millipore) to discard cell debris. The TCM supernatant was concentrated to 200 μL by ultrafiltration with the use of a Millipore 3 kDa Centricon column. The TCM was stored in aliquots at -80 °C until use. The TCM loading volume was adjusted according to the number of living cells in each sample.

### Capillary tube formation assay

HUVECs (2.4×10^4^) were grown in the TCM for 12 hours at 37 °C in a 48-well plate coated with Matrigel (354230, Corning). The formation of capillary-like structures was captured by a light microscope. The branch points of the formed tubes, which represented the ability of angiogenesis, were counted in three low-power fields (100×).

### Chicken chorioallantoic membrane (CAM) assay

CAM assay was performed according to a protocol previously described 23. Briefly, a 1-cm-diameter window was opened in the shell of each egg with a 7-day-old chicken embryo (Yueqin Breeding Co.). The surface of the dermic sheet on the floor of the air sac was removed to expose the CAM. First, a 0.5-cm-diameter filter paper was placed on top of the CAM, and 200 μL of TCM was added onto the center of the paper. After the window was closed with sterile adhesive tape, the eggs were incubated at 37 °C under 80% relative humidity for 48 h. Following fixation with a stationary solution (methanol/acetone, 1:1) for 15 min, the CAMs were cut and mounted on slides. Gross photos of each CAM were taken under a digital camera. The effect of TCM was evaluated by counting the number of second-and third-order vessels.

### *In vitro* cell migration assay

*In vitro* cell migration assays were performed in Transwell chambers (8 µm pore size; Costar) according to the manufacturer's instructions. 2×10^4^ cells were placed into the top chamber of each insert (BD Biosciences) and were incubated at 37 °C for 8 h.

### Cell proliferation assay

Two experiments were performed to analyze the proliferation of HUVECs. In the CCK-8 assay, HUVECs cells were seeded at 500 cells per well in 96-well microplates for 12 h and then, the complete medium was replaced with TCM and cultured for the indicated hours. Cell proliferation was measured using the Cell Counting Kit-8 (CCK-8) assay kit (Dojindo Corp.) according to the manufacturer's instructions. In another proliferation assay, HUVECs (6×10^5^) were grown in complete medium for 12 h at 37 °C in a 6-well plate, and then replace the complete medium with TCM and cultured for another 24 h. The cells were trypsinized and counted using the Scepter^TM^ Handheld Automated Cell Counter (Millipore). Three independent experiments were performed for this step.

### Animal studies

Five-week-old male nude mice were used for subcutaneous xenograft model. Hepatoma cells were suspended in 50 µL of Dulbecco's Modified Eagle Medium (DMEM) and Matrigel (1:1), and were then injected subcutaneously into either side of the posterior flank of the same male BALB/c athymic nude mice. Eight nude mice were included and their tumor growth was examined during the following 30 days. The mice were sacrificed and the tumors were dissected, fixed in formalin, and embedded in paraffin. All experimental procedures involving animals were performed according to the *Guide for the Care and Use of Laboratory Animals* (NIH publications Nos. 80-23, revised 1996), and in accordance with the institutional ethical guidelines for animal experiments.

### Luciferase reporter assay

To explore the effect of miR-130b-3p on the HOXA5 3'-UTR, HEK293T cells in a 6-well plate were co-transfected with 10nM of miR-130b-3p or NC duplex, 1 µg pEZX-HOXA5-3'-UTR-WT or pEZX-HOXA5-3'-UTR-MUT and cultured for 24 h. The cells were then trypsinized and transferred to a 96-well plate. After 24 hours, a luciferase reporter assay was performed following the manufacturer's instructions. Renilla luciferase activity was normalized to firefly luciferase activity.

To dissect the promoter region of miR-130b-3p, Huh7 and MHCC-97H cells were transfected with firefly luciferase reporter plasmids in a 48-well plate. To explore the effect of Sp1 knockdown on the miR-130b-3p promoter activity, Huh7 and MHCC-97H grown in a 48-well plate were co-transfected with siSp1 duplex and firefly luciferase reporter. Cell lysates were collected 48 h after the transfection and were subjected to luciferase activity assays using the luciferase reporter system (Genecopoeia). The data are presented as the mean ± standard error of the mean (SEM) from at least three independent experiments.

### RNA isolation, quantitative real-time PCR, and western blot

Total RNA was extracted from the HCC tissues and cell lines using Trizol reagent kit (Life Technologies, Carlsbad, CA). After treatment with DNase I (TaKaRa, Dalian, China), 2 µg of total RNA was used for cDNA synthesis with random hexamers and Superscript III (Invitrogen). The cDNA templates were subjected to PCR amplification.

qRT-PCR analysis of miR-130b-3p was performed on an ABI PRISM 7900 Sequence Detector using an SYBR Green PCR Kit (Applied Biosystems, Carlsbad, CA). The following primers were used for detection: HmiRQP0159 for miR-130b-3p, HmiRQP9001 for U6 (Genecopoeia, Guangzhou, China). All reactions were run in triplicate. The cycle threshold (Ct) values should not differ more than 0.5 among triplicates. The miR-130b-3p level was normalized to RNU6B, which yielded a 2^- ΔΔCt^ value.

Equal amounts of cell protein lysates were separated in 10% SDS-polyacrylamide gels and electrophoretically transferred to polyvinylidene difluoride membranes (Millipore), then detected with rabbit polyclonal antibody specific for HOXA5 (Sigma-Aldrich) and a commercial ECL kit (Pierce). Protein loading was estimated using a mouse anti-β-actin monoclonal antibody (Sigma-Aldrich).

### Immunohistochemical staining

Paraffin-embedded tissue sections from nude mice or HCC patients were applied to IHC using a rabbit Ab against human CD31 (cat. ab28364, Abcam), CD34 (cat. ab81289, Abcam), VEGF (cat. ab46154, Abcam) or HOXA5 (cat. HPA029319, Sigma-Aldrich), respectively. Immunoreactivity for VEGF and HOXA5 proteins was scored using a semi-quantitative method by evaluating the number of positive tumor cells over the total number of tumor cells. Scores were assigned by using 5% increments (0%, 5%, 10% till 100%), as described in a previous study 24. Any discrete cluster or single cell stained for CD31 or CD34 was considered as one microvessel. Five representative fields were counted, and the average number of microvessels per field (×200) is presented 25.

### Chromatin immunoprecipitation (ChIP) assay

ChIP was performed using the SimpleChIP Enzymatic Chromatin IP kit (Cell Signaling Technology). HCC cells were crosslinked with formaldehyde, lysed with SDS buffer followed by ultrasonication, then, incubated with specific antibodies or normal mouse IgG. After washing with high salt and low salt, DNA was eluted and de-crosslinked, and enrichment was examined using PCR.

### Statistical analysis

Survival curves were computed using the Kaplan-Meier method and analyzed by the log-rank test. Significant prognostic factors found by univariate analysis (p < 0.05) were entered into a multivariate analysis using the Cox proportional hazards model. The Fisher test was used to analyze the association of HOXA5 expression with the investigated patients' clinicopathological factors. The Student *t* test or the Mann-Whiney *U* test was used to compare the values between subgroups. Data were expressed as mean ± SD. The program Statistical Package for Social Science version 22 (SPSS Inc., Chicago, IL, USA) and R statistical package (R software version 3.4.1; R Foundation for Statistical Computing, Vienna, Austria) was used for all analyses. A p value < 0.05 was considered statistically significant.

## Results

### miR-130b-3p is upregulated in HCC and predicts poor prognosis

**Figure 1:**
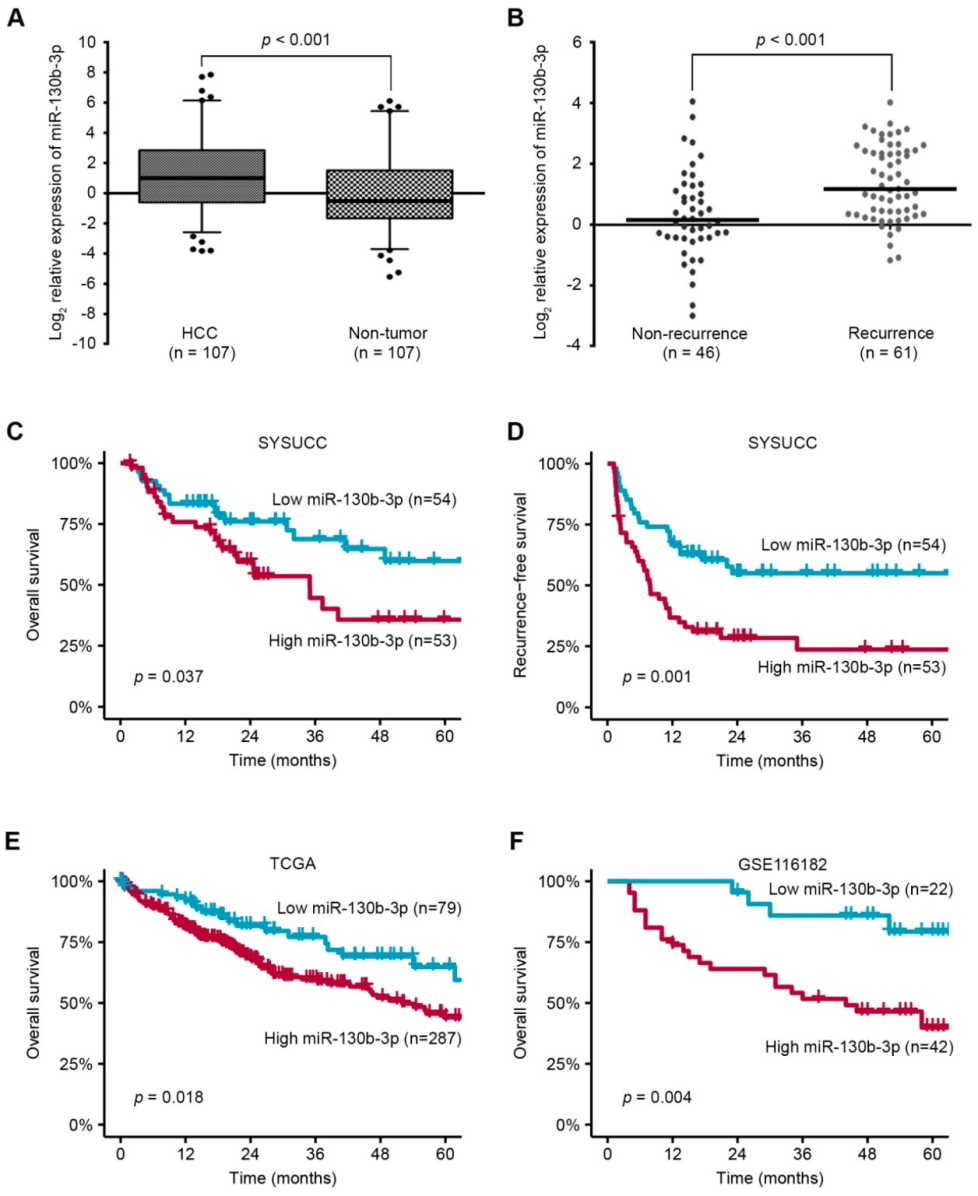
miR-130b-3p is upregulated in HCC and predicts poor prognosis. (A) The expression of miR-130b-3p in HCC is markedly upregulated, compared with adjacent non-tumor liver tissues. (B) The expression of miR-130b-3p is higher in tumor samples of recurrent HCC patients. (C) The Kaplan-Meier method reveals that higher miR-130b-3p level is associated with shorter overall survival of HCC patients. (D) The Kaplan-Meier method reveals that higher miR-130b-3p level is associated with shorter recurrence-free survival of HCC patients. (E) Kaplan-Meier analyses of the correlation between miR-130b-3p levels and the overall survival in the TCGA HCC cohort. (F) Kaplan-Meier analyses of the correlation between miR-130b-3p levels and the overall survival in GEO database (GSE116182).

Using miRNA microarray, we found that miR-130b-3p was up-regulated in HCC 15. To validate the expression of miR-130b-3p, we quantified the level of miR-130b-3p in 107 paired HCC and adjacent non-tumor liver tissues (Cohort 1). Notably, the expression of miR-130b-3p in HCC was markedly increased, compared with adjacent non-tumor liver tissues (p < 0.001) (Figure 1A). Moreover, the expression of miR-130b-3p in patients with recurrence was higher than those without recurrence (p < 0.001) (Figure 1B).

Next, we investigated whether the overexpression of miR-130b-3p in HCC tissue correlated with the clinicopathological features and prognosis of the HCC patients. An association between the increased miR-130b-3p expression and increased tumor size was observed (Table S1). The Kaplan-Meier analyses revealed that higher miR-130b-3p level was associated with both shorter overall survival and recurrence-free survival of the HCC patients (p = 0.037 and 0.001, respectively) (Figure 1C-D), and was also confirmed using the TCGA cohort and GEO data set (GSE116182) (p = 0.018 and 0.004, respectively) (Figure 1E-F). Notably, multivariate analyses confirmed that overexpressed miR-130b-3p level was an independent predictor for both shorter RFS and OS in HCC patients (p <0.001 and p = 0.047, respectively) (Table S2). Collectively, these data suggest that an overexpression of miR-130b-3p may contribute to the progression of HCC and predict poor prognosis of HCC patients.

### miR-130b-3p enhances the capacity of liver cancer cells to promote angiogenesis

**Figure 2:**
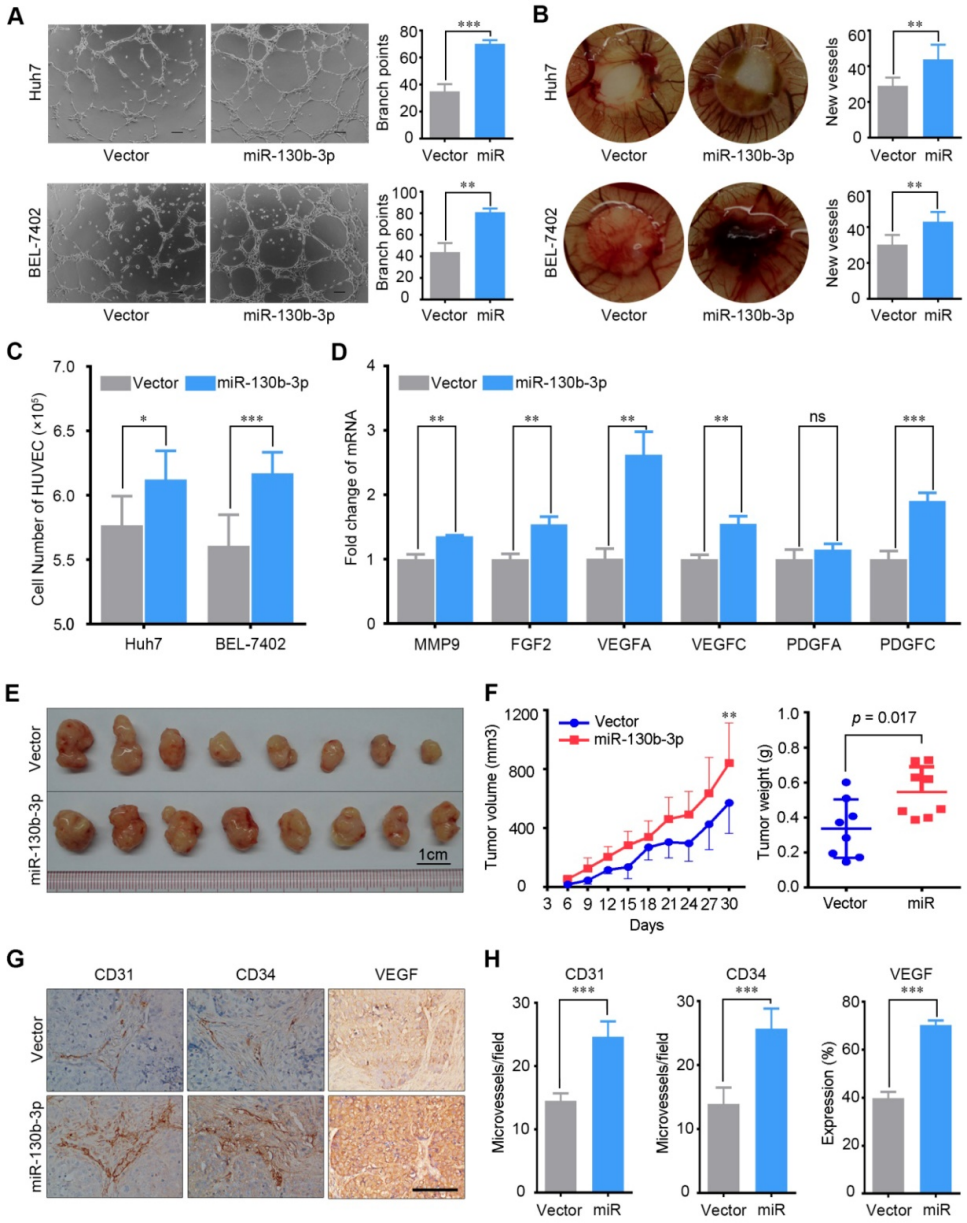
miR-130b-3p promotes tumor angiogenesis *in vitro* and *in vivo*. (A) TCM from miR-130b-3p transfected HCC cells induced tube formation of HUVECs. Representative images of capillary-like structures and the number of branch points of HUVECs are shown. (B) Effect of miR-130b-3p on vascularization in the CAM angiogenesis model. Filter discs soaked with TCM were loaded on the CAMs of Day 8 chick embryos. After incubation for 5 days, the area under and surround the filter was fixed and photographed. Representative images of neovascularization and the number of new blood vessels are shown. (C) HUVECs proliferation was promoted by the TCM from miR-130b-3p transfected HCC cells. HUVEC cells were seeded on 6-well plate with a density of 3 × 10^5^ cells per well and were cultured with SFM supplemented with 20% FBS and 0.3% EGF for 6 hours and shifted to TCM from indicated cells and cultured for additional 24 h. The number of HUVEC cells was counted using the ScepterTM Handheld Automated Cell Counter. Results were based on six independent experiments. (D) The mRNA level of angiogenesis relevant genes was upregulated by miR-130b-3p. The expression of MMP9, FGF2, VEGFA, VEGFC, PDGFA, PDGFC in Huh7 cells transfected with miR-130b-3p or vector were determined by qRT-PCR. Results were based on at least three independent experiments. Data are presented as their mean ± SD. (E) miR-130b-3p overexpressed and control BEL-7402 cells were subcutaneously injected into nude mice (n = 8). The nude mice were sacrificed on day 30 after inoculation and the tumor were harvested. (F) The tumor weight on day-30 after inoculation are presented (left). The tumor growth curve was measured every 3 days for 30 days after inoculation. The mean tumor volume was significantly increased in the miR-130b-3p overexpression group (right). (G) Representive immunohistochemical images of endothelial markers CD31, CD34 and VEGF on serial sections of tumor samples are shown. Scale bar, 100 µm. (H) The staining intensity of CD31, CD34, and VEGF from tumor samples are shown. Data are presented as their mean ± SD. Statistical analysis was performed using the Student's t-test. * p < 0.05, **p < 0.01, ***p < 0.001.

To evaluate the role of miR-130b-3p in the angiogenesis of HCC, *in vitro* capillary tube formation assay and chicken chorioallantoic membranes (CAMs) assay were performed. Huh7 and BEL-7402 with low endogenous miR-130b-3p expression were stably transfected with miR-130b-3p (Figure S1A-B). The conditioned medium of stable cell lines was collected and supplied to the culture medium for HUVECs. We observed that HUVECs cultured with the conditioned medium derived from the miR-130b-3p overexpressed cell lines developed more capillary-like structures and branch points; implying the pro-angiogenesis function of miR-130b-3p in HCC (Figure 2A). Besides, the CAMs assay also revealed that ectopic expression of miR-130b-3p strongly facilitated the formation of second- and third-order micro-vessels (Figure 2B). Moreover, we observed that the TCM of miR-130b-3p- transfectants could promote HUVECs proliferation (Figure 2C). Finally, the angiogenesis related genes, including MMP9, FGF2, VEGFA, VEGFC, PDGFA and PDGFC, were found to be upregulated in miR-130b-3p overexpressed HCC cells (Figure 2D). On the other hand, HUVECs treated with the TCM of anti-miR-130b-3p- transfectants had less capacity for angiogenesis (Figure S2). In summary, these findings suggest that miR-130b-3p could enhance the pro-angiogenesis capacity of HCC cells *in vitro*.

To further assess the *in vivo* effects of miR-130b-3p overexpression on angiogenesis, miR-130b-3p-BEL-7402 cells and control cells were subcutaneously implanted into nude mice. At 9 days post injection, the mean volumes of xenograft tumors generated from the miR-130b-3p-BEL-7402 cells were markedly larger than those originating from the control cells (n = 8 animals per group, p < 0.01) (Figure 2F, left panel). At 30 days post injection, the mice were sacrificed and the tumors were weighted. The mean tumor weight of the miR-130b-3p-BEL-7402 group was greater than the control group (p = 0.017) (Figure 2F, right panel).

Next, the microvessel density of the tumors was analyzed by immunohistochemical staining using CD31 and CD34 antibodies. Both of them showed a significant increase of microvessel density in tumors with miR-130b-3p overexpressed as compared to the controls (p < 0.001). Besides, the expression of VEGF was also found to be upregulated in miR-130b-3p overexpressed tumors. These results clearly showed that miR-130b-3p was involved in promoting tumor angiogenesis in HCC (Figure 2G-H).

### HOXA5 is a direct target gene of miR-130b-3p

The potential targets of miR-130b-3p were predicted by the following five bioinformatics algorithms, targetScan, picTar, RNA22, PITA and miRanda. The intersections were retrieved by starbase V2.0 26 and thirty-one genes were found to be potential targets (Table S6). The top 5 genes, JARID2, DCBLD2, E2F7, HOXA5, and PHF3, were screened by qRT-PCR. Among them, HOXA5 was found to be the most significantly downregulated target in HCC cells transfected with miR-130b-3p (Figure S3). As transcription factors encoded by the homeobox genes have a pivotal role in governing the process of tumor angiogenesis, we decided to explore the function of HOXA5.

**Figure 3:**
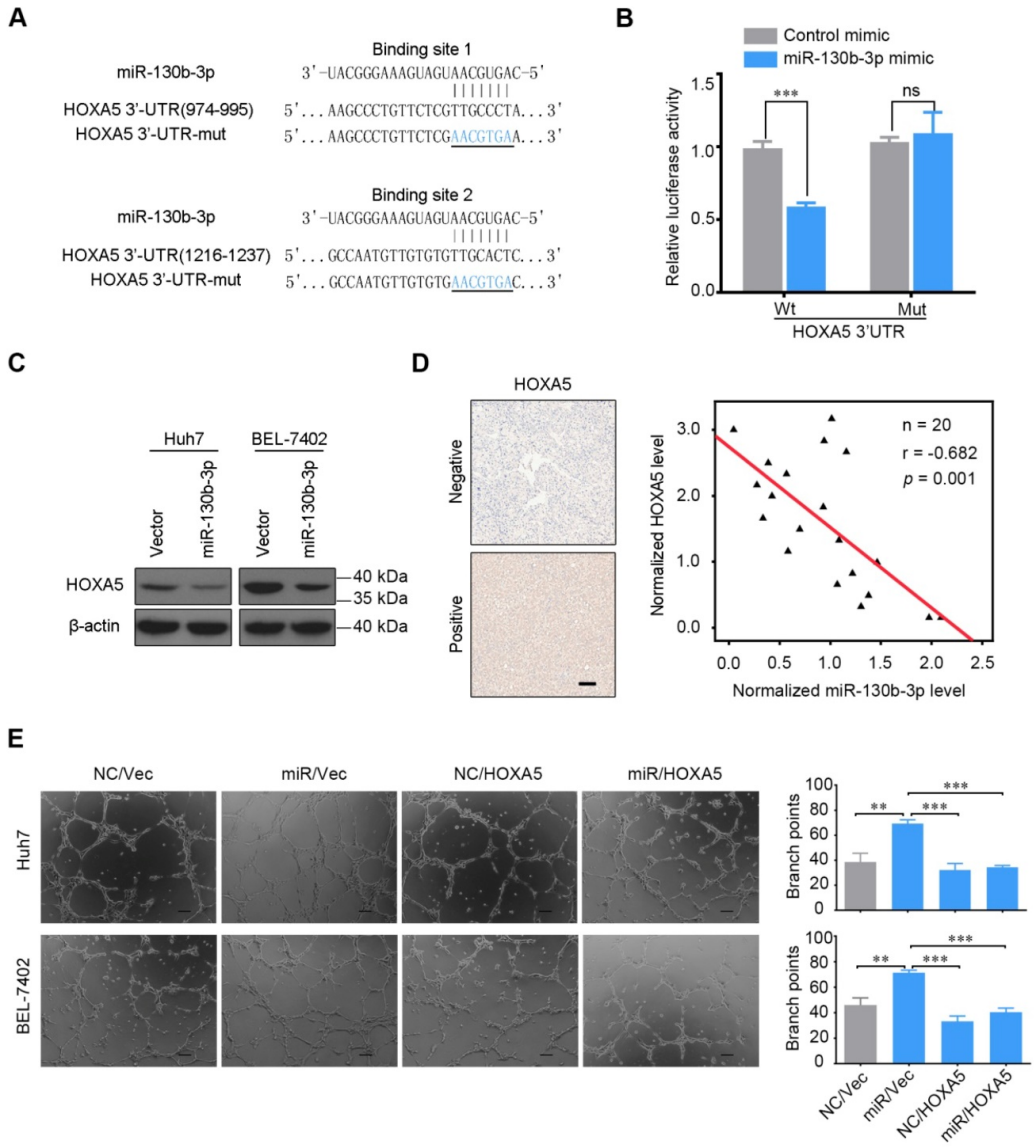
HOXA5 is a direct target of miR-130b-3p. (A) Luciferase reporter plasmids were inserted with wild-type or mutant HOXA5 3'-UTR immediately downstream of the luciferase gene. The sequences of two predicted miR-130b-3p binding sites within the HOXA5 3'-UTR, including wild-types and mutants (underlined) are shown. (B) Relative luciferase activity was analyzed in HEK-293T cells transfected with indicated reporter plasmids and miR-130b-3p mimic or control. (C) miR-130b-3p overexpression downregulated endogenous HOXA5 protein level in Huh7 and BEL-7402 cells. (D) HOXA5 protein level negatively correlated with miR-130b-3p in HCC. Left, representative immunohistochemical images of HOXA5 in HCC are shown (magnification ×200). Right, the correlation between miR-130b-3p and HOXA5 protein level in HCC tissues are shown (n = 20). miR-130b-3p was detected by qRT-PCR and normalized to U6 expression. Statistical analysis was performed using the Spearman's correlation coefficient. (E) HOXA5 partially impaired the miR-130b-3p induced angiogenesis phenotype. HCC cells were co-transfected with miR-130b-3p mimic or control mimic and pEZ-HOXA5 or vector and the TCMs were collected 24 h after transfection. HUVECs were seeded above the Matrigel basement membrane matrix (growth factor reduced) and cultured with TCM for 8 h. Representative images of capillary-like structures (Left) are shown. Quantification of tube formation (Right) was performed basing on the number of branches. Results were based on at least three independent experiments.Data are presented as their mean ± SD. Statistical significance was determined by Student's t-test. * p < 0.05, **p < 0.01, ***p < 0.001.

Two conserved binding sites for miR-130b-3p were identified in 3'UTR region of the HOXA5 gene. To elucidate whether HOXA5 was directly regulated by miR-130b-3p, we constructed a luciferase reporter plasmid containing the 3'UTR of HOXA5. As shown in Figure 3B, the relative luciferase activity of the reporter containing the wild-type 3'UTR was significantly decreased when co-transfected with miR-130b-3p mimics. On the contrary, the luciferase activity of the mutant-type 3'UTR was similar between the miR-130b-3p mimics and control mimics. As shown in Figure 3C, the protein expression of HOXA5 was significantly decreased after the overexpression of miR-130b-3p. These indicated that HOXA5 was directly regulated by miR-130b-3p.

To explore the correlation between miR-130b-3p and HOXA5, the expression of miR-130b-3p and HOXA5 in HCC tissues were detected by qRT-PCR and immunohistochemistry, respectively. After normalization, the expression of miR-130b-3p and HOXA5 were analyzed by Pearson's correlation coefficient analysis. Interestingly, the HOXA5 protein level was inversely correlated with miR-130b-3p expression level (Figure 3D). This implied that endogenous HOXA5 in HCC were negatively regulated by miR-130b-3p.

Furthermore, we evaluated the role of HOXA5 in miR-130b-3p-mediated phenotypes. We found that the overexpression of HOXA5 in miR-130b-3p-transfectants attenuated the pro-angiogenic effect of miR-130b-3p (Figure 3E). These data suggested that HOXA5 acted as a direct functional target gene of miR-130b-3p.

### Down-regulation of HOXA5 promotes tumorigenicity and angiogenesis in HCC

**Figure 4:**
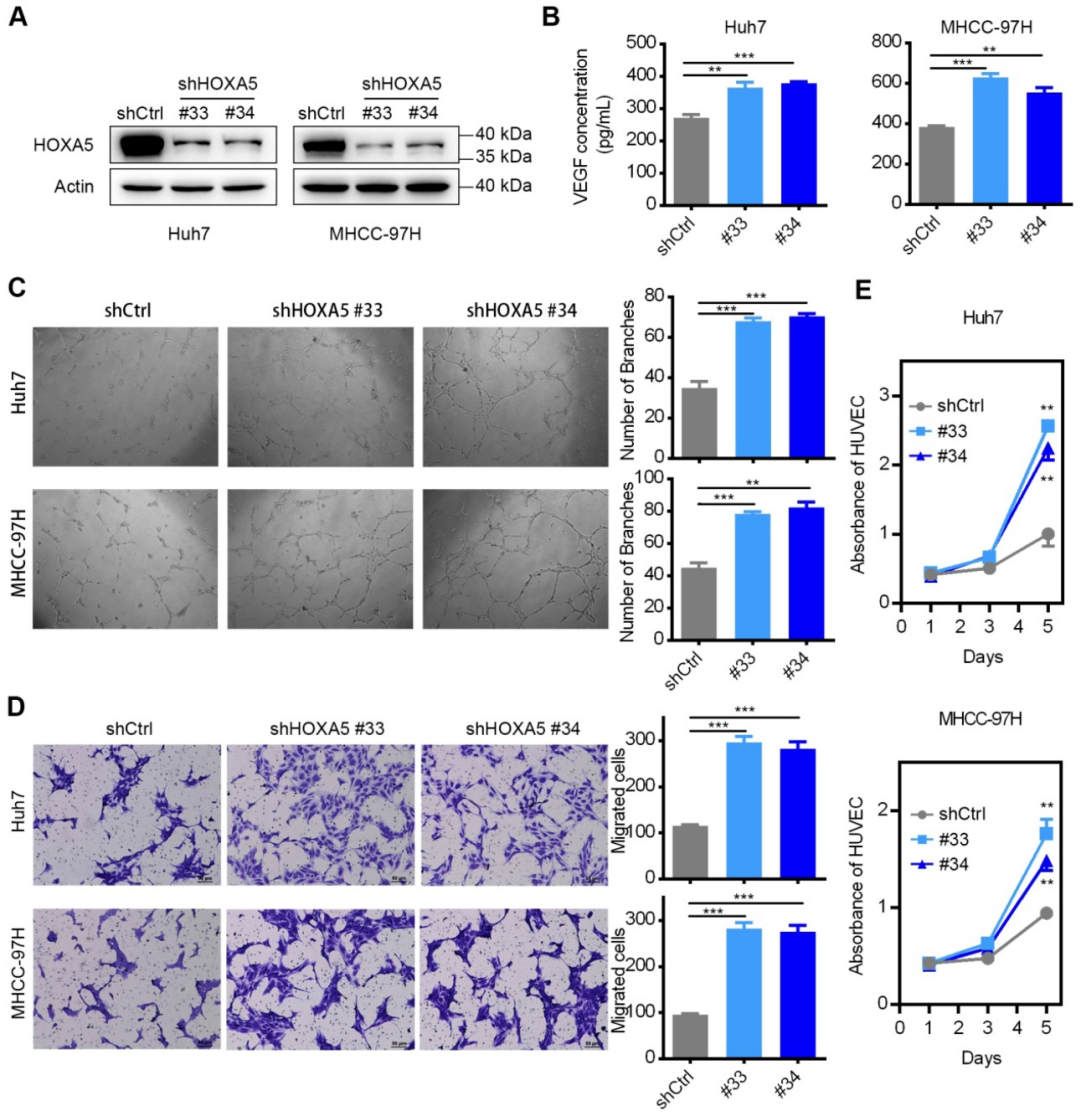
The down-regulation of HOXA5 promotes angiogenesis in HCC *in vitro*. (A) The knockdown efficiency of shHOXA5 in HCC cells by Western Blot (Huh7 and MHCC-97H). (B) The down-regulation of HOXA5 increased the levels of secreted VEGF in HCC cells. VEGF was measured by ELISA in the supernatants of indicated HCC cells. (C) The down-regulation of HOXA5 enhanced HCC cell-induced tube formation of HUVECs. HUVECs were cultured in the presence of 75% TCM from Huh7 or MHCC-97H cells transfected with indicated plasmids. Representative images of tube formation and the number of branch points of HUVECs are presented. (D) Endothelial recruitment assay revealed an enhanced effect of HOXA5 down-regulation on HCC cell-induced HUVECs migration. HUVECs were seeded in the upper transwell chambers with TCM in the lower compartments and incubated for 12 h. (E) The down-regulation of HOXA5 promoted HCC cell-induced HUVECs proliferation. HUVECs were grown in complete medium for 12 h at 37 °C in a 96-well plate and then replace the complete medium with TCM and cultured for an indicated period of time. Cell viability was measured using the Cell Counting Kit-8 (CCK-8) assay kit (Dojindo Corp.) according to the manufacturer's instructions. Three independent experiments were performed. *p < 0.05; **p < 0.01.

To explore the anti-oncogenic role of HOXA5 in HCC, two shRNAs (shHOXA5 #33 and shHOXA5 #34) specially targeting HOXA5 were transfected into Huh7 and MHCC-97H, respectively. The silencing effect was validated by western blotting analysis, and the results revealed that both shRNAs could specifically down-regulate the expression of HOXA5 (Figure 4A). Since VEGF is a pivotal activator of angiogenesis-related pathways, we examined the VEGF level in the TCM. ELISA assay revealed that the VEGF levels were significantly higher in the shHOXA5 groups (Figure 4B). To explore the biological significance of HOXA5 in tumor angiogenesis, *in vitro* tube formation assays were performed. TCMs from tumor cells were supplied to the culture medium for HUVECs. We observed that the HUVECs treated with TCM derived from the shHOXA5-transfectants developed more capillary-like structures compared to cells treated with TCM derived from scramble shRNAs-transfectants (Figure 4C). In addition the TCM from shHOXA5-transfectants significantly promoted the proliferation and migration of HUVECs, compared to the corresponding controls (Figure 4D-E). Moreover, the angiogenesis-related genes were upregulated in HCC cells transfected with shHOXA5 (Figure S4E-F). However, the ectopic expression of HOXA5 had little impact on tumor angiogenesis (Figure S4C-D, and Figure S5).

**Figure 5:**
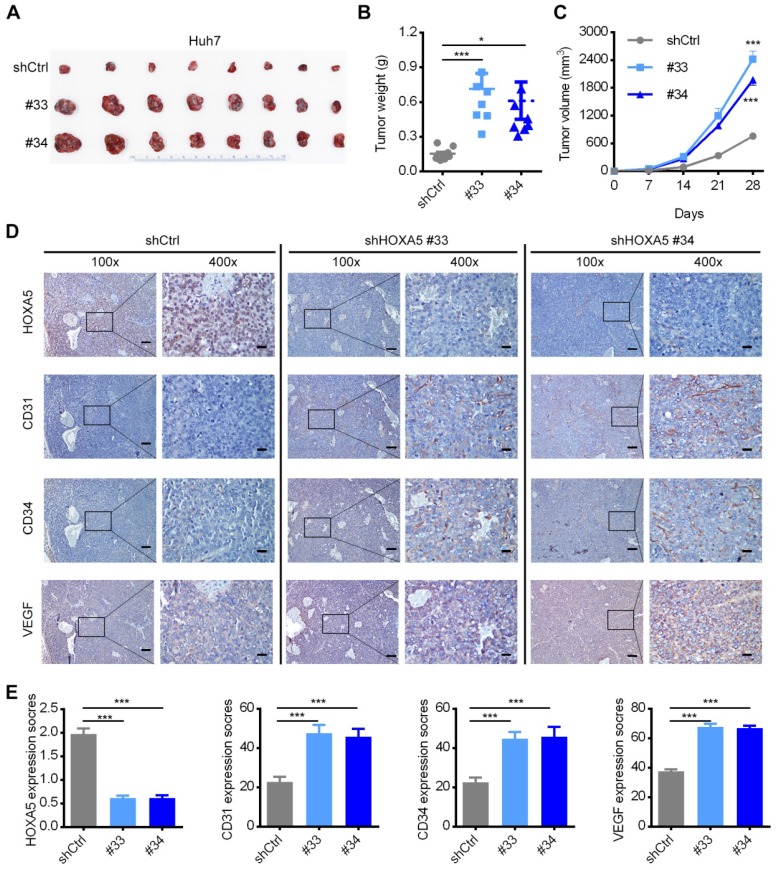
The down-regulation of HOXA5 promotes HCC tumorigenicity and angiogenesis *in vivo*. (A) HOXA5 down-regulation promoted the growth of Huh7 xenograft tumors. HOXA5 down-regulated and control Huh7 cells were subcutaneously injected into nude mice (n = 8). The nude mice were sacrificed on day-28 after inoculation and the tumor were harvested. (B) The tumor weight on day-28 after inoculation are presented. (C) The tumor growth curve was measured every week after inoculation. The mean tumor volume was significantly increased in the HOXA5-downregulated group. (D) HOXA5 down-regulation elevated the expression of angiogenesis markers. Immunohistochemical expression of angiogenesis markers CD31, CD34 and VEGF were detected on serial sections of tumor samples. Scale bar, 100 µm (100x), 25 µm (400x). (E) The staining intensity of CD31, CD34, and VEGF from tumor samples were shown.Data are presented as their mean ± SD. Statistical analysis was performed with Student's t-test. *p < 0.05; ***p < 0.001.

To further verify the *in vivo* anti-oncogenic role of HOXA5, we conducted subcutaneous injection of HCC cells into nude mice, which were then sacrificed after four weeks for collecting the tumoral tissues. As shown in Figure 5A-C, both the tumor size and weight were significantly higher in the Huh7-shHOXA5 group than in Huh7-shCtrl group. IHC staining of the tumoral tissues showed markedly lower HOXA5 level and higher CD31, CD34 and VEGF levels in the Huh7-shHOXA5 group than in the Huh7-shCtrl group (Figure 5D-E). Collectively, these data proved that HOXA5 may suppress angiogenesis in HCC.

### Down-regulation of HOXA5 predicts poor prognosis and negatively correlates with angiogenesis markers

**Figure 6:**
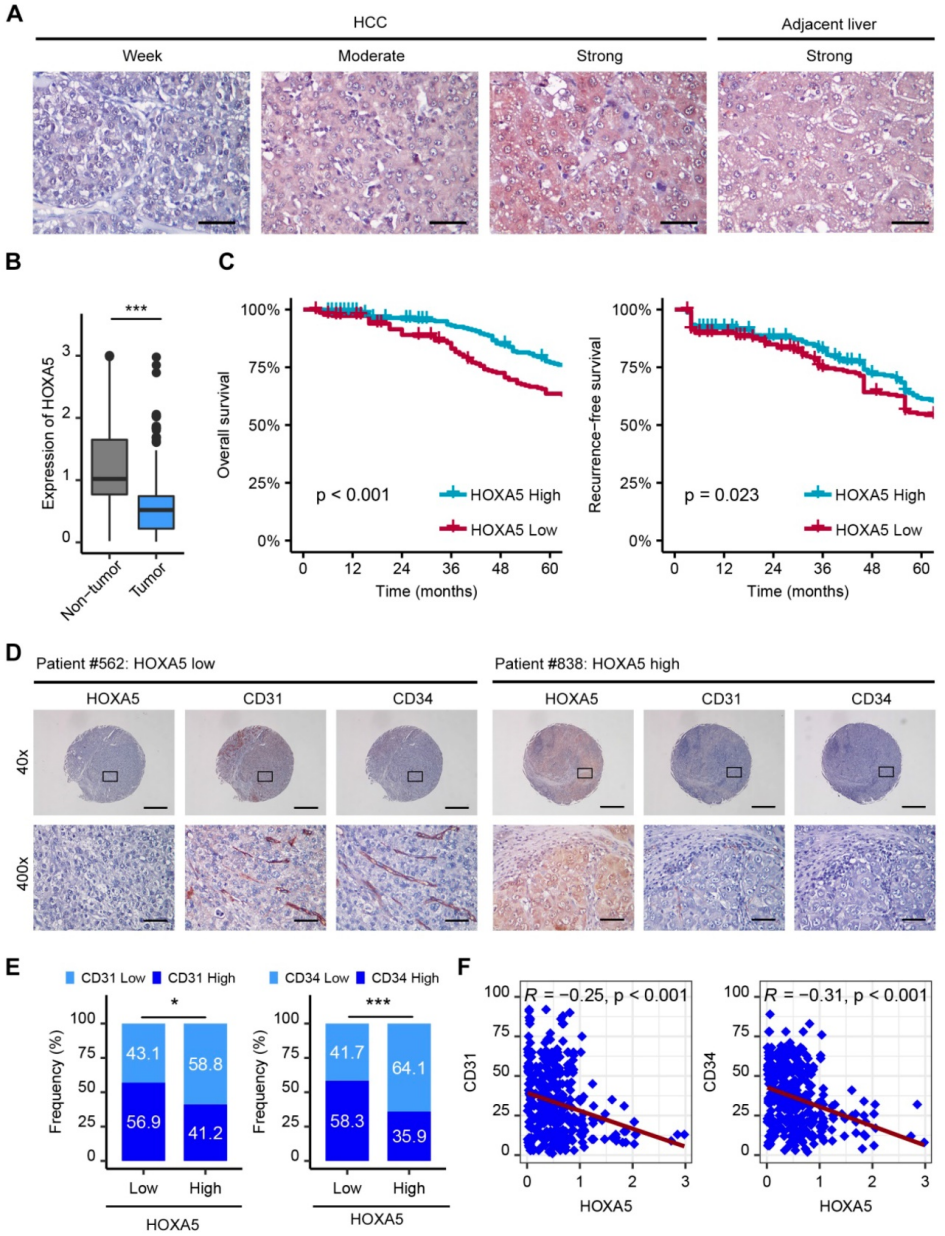
The down-regulation of HOXA5 predicts poor prognosis and negatively correlates with angiogenesis in HCC. (A) Representative IHC staining images of HOXA5 in HCC and adjacent non-tumor liver tissues. Scale bar, 500 μm. (B) HOXA5 expression was significantly decreased in HCC tissues. Quantification of HOXA5 levels according to IHC scores. (C) Kaplan-Meier plot revealed shorter overall and recurrence-free survival in the lower HOXA5 group. Based on the minimum *p*-value approach, the 49th percentile of the HOXA5 level in 449 HCC tissues was chosen as the cut-off value for dividing the HOXA5-low level group (n = 220) from the HOXA5-high level group (n = 229). (D) (E) and (F) HOXA5 was negatively correlated with angiogenesis markers CD31, CD34 in HCC. (D) Representative staining images of HOXA5 and CD31, CD34 are shown. (E) The expression of CD31 and CD34 in HOXA5-low and HOXA5-high group, respectively. The median IHC scores were chosen as the cut-off value for separating the low and high groups (n = 314). (F) Correlations between HOXA5 and CD31, CD34 in HCC were assessed using the Pearson correlation and linear regression analytics (n = 314). * p < 0.05, *** p < 0.001.

The protein level of HOXA5 in 449 human HCC samples and matched adjacent nontumor liver tissues were detected by immunohistochemistry (IHC). The results revealed that HOXA5 was significantly down-regulated in HCC tissues (Figure 6A-B). Clinicopathological analyses revealed that HOXA5 was negatively correlated with tumor size and AFP (Table S3). Further survival analysis showed that the low HOXA5 group had shorter overall survival and recurrence-free survival than the high HOXA5 group (Figure 6C). Besides, the prognostic value of HOXA5 in HCC was also confirmed in the GEO data set (Figure S6A-B). Multivariate Cox regression analysis further demonstrated that low HOXA5 expression was an independent predictive indicator for overall survival (hazard ratio [HR] 1.758, 95% confidence interval [CI], 1.295-2.386; p < 0.001) and recurrence-free survival (hazard ratio [HR] 1.625, 95% confidence interval [CI], 1.239-2.132; p < 0.001) in HCC (Table S4). Moreover, the expression levels of CD31 and CD34 in tumor specimens from 314 HCC patients were also detected by IHC staining, to investigate the correlation between HOXA5 and MVD. These patients were divided into two groups, namely the HOXA5_high group (n = 170) and the HOXA5_low group (n = 144) based on the HOXA5 expression.

We found that the rate of strong CD31 staining was higher in the HOXA5_low group, compared to that in the HOXA5_high group (82/144, 56.9% vs. 70/170, 41.2%, p < 0.05) (Figure 6E, left panel). Consistently, the rate of strong CD34 staining was also higher in the HOXA5_low group (84/144, 58.3%) than in the HOXA5_high group (61/170, 35.9%, p < 0.001) (Figure 6E, right panel). We also found significant negative correlations between HOXA5 and CD31 (*R* = -0.25, p < 0.001), as well as CD34 in HCC tissues (*R* = -0.31, p < 0.001) (Figure 6F). Consistently, the correlations between HOXA5 and CD31/CD34 were also validated in the GEO data set (Figure S6C-D).

### Sp1 regulates miR-130b-3p expression through miR-130b-3p promoter binding

To determine the mechanism of miR-130b-3p up-regulation in HCC, the AliBaba2.1 (http://www.gene-regulation.com/pub/programs/alibaba2/) software was used to search for predicted transcription binding sites. Sixty three transcription factors which may bind to the promoter of miR-130b-3p were predicted (Table S7). The top 3 transcription factors, which had the most potential binding sites, were Sp1 (97 sites), C/EBPalpha (11 sites) and NF-1 (10 sites). A previous study reported that Sp1 may bind to the putative miR-130b-3p promoter sequence, as predicted by TSSG promoter prediction program 27. Moreover, since Sp1 is considered as a basal transcription factor and regulates angiogenesis in multiple tumors 28-30, we thereby chose Sp1 for further analysis.

**Figure 7:**
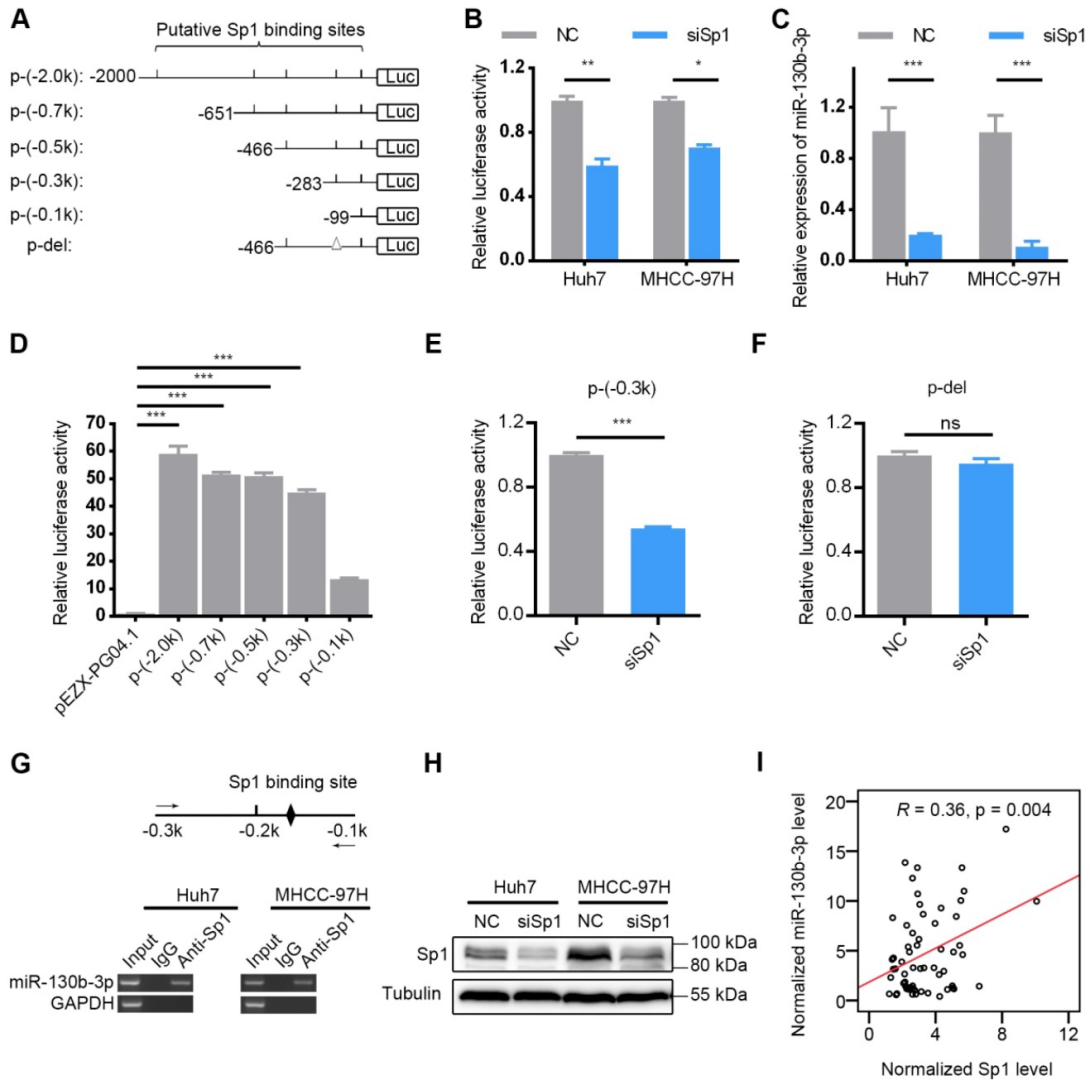
Sp1 is essential for miR-130b-3p transcription. (A) Schematic diagram of firefly luciferase reporter constructs containing the indicated genomic segments upstream of miR-130b-3p gene. Putative Sp1 binding sites are depicted as short vertical lines. Deletion of the Sp1 binding site is depicted as triangle (Δ). (B) Silencing of Sp1 decreased the miR-130b-3p promoter activity. HCC cells were reversely transfected with NC or siSp1 for 24 h, followed by transfection with p-(-2.0 k) for another 48 h before luciferase activity analysis. (C) Silencing of Sp1 reduced miR-130b-3p level. The indicated HCC cells were transfected with NC or siSp1 for 48 h before qRT-PCR analysis. (D) Effect of fragment deletions on miR-130b-3p promoter activity. Huh7 cells were transfected with the indicated luciferase reporter for 48 h before luciferase activity analysis. The mean value of pEZX-PG04.1-transfectant from multiple independent experiments was set as control. (E) Silencing of Sp1 reduced the promoter activity of p-(-0.3 k). (F) Silencing of Sp1 did not change the activity of p-del. In (E) and (F), Huh7 cells were reversely transfected with negative control (NC) or siSp1 duplex for 24 h and then transfected with the indicated luciferase reporter for another 48 h. The mean value of NC-transfectant from multiple independent experiments was set as control. (G) Sp1 interacted with the miR-130b-3p promoter in HCC cells. Scheme of the amplicon is shown on top. The putative Sp1 binding site located at the -0.3 to -0.1-kb region is illustrated as a black diamond. The solid arrows represent primers used for semi-quantitive PCR. ChIP assays detected a specific band of the expected size in the input DNA and the Sp1 antibody (Anti-Sp1)-precipitated DNA. Genomic region upstream of GAPDH was used as a negative control. (H) Certification of knockdown efficiency of siSp1 in HCC cells (Huh7 and MHCC-97H). (I) The mRNA level of Sp1 positively correlated with miR-130b-3p in HCC specimens (n = 62). Sp1 and miR-130b-3p was detected by qRT-PCR, and normalized to actin and U6, respectively. Statistical analysis was performed using Pearson correlation coefficient. ns, not significant; * p < 0.05; ** p < 0.01; *** p < 0.001.

The miR-130b-3p upstream region (-1 to -2000kb) was analyzed by JASPAR (http:// http://jaspar.genereg.net/), and six binding sites of Sp1 were predicted in the putative promoter region (Figure 7A), using a cut-off score of 10 and a relative profile score threshold of 80% (Table S8). We used RNA interference to silence the Sp1 expression (Figure 7H). Our findings showed that silencing Sp1 resulted in a significant decrease in the promoter activity of p-(-2.0k) (Figure 7B) as well as the endogenous miR-130b-3p level (Figure 7C). To further confirm the functional Sp1 binding sites within the putative promoter region, a 5' deletion analysis was conducted (Figure 7A). Compared with the promoter activity of p-(-2.0k), no significant change in p-(-0.7k), p-(-0.5k) or p-(-0.3k) was detected and a remarkably decreased activity in the promoter activity of p-(-0.1k) was observed (Figure 7D). These results indicated that the -0.3 to -0.1kb region was crucial for miR-130b-3p transcription. Between the -0.3 to -0.1kb region, there was only one Sp1 potential binding site (Figure 7A). Sp1 silencing dramatically reduced the activity of p-(-0.3k) (Figure 7E) but had no effect on that of p-del without Sp1 binding site within the -0.3 to -0.1kb region (Figure 7F).

In addition, ectopic expression of Sp1 did not change the promoter activity of p-(-2.0 k) (Figure S7B). Silencing of Sp1 expression reduced the promoter activity of p-(ΔA) and p-(ΔB) (Figure S7C-D). To determine whether Sp1 bounded directly to the miR-130b-3p promoter, we performed chromatin immunoprecipitation (ChIP) assay with anti-Sp1 antibody, and the result demonstrated an interaction between Sp1 and the miR-130b-3p promoter (Figure 7G). Furthermore, the expression of Sp1 was found to be positively correlated with miR-130b-3p in HCC specimens (Figure 7I). These data collectively demonstrated that Sp1 transcriptionally regulated miR-130b-3p expression in HCC cells.

### Down-regulation of HOXA5 promotes angiogenesis in HCC via the PI3K/AKT/mTOR signaling pathway

**Figure 8:**
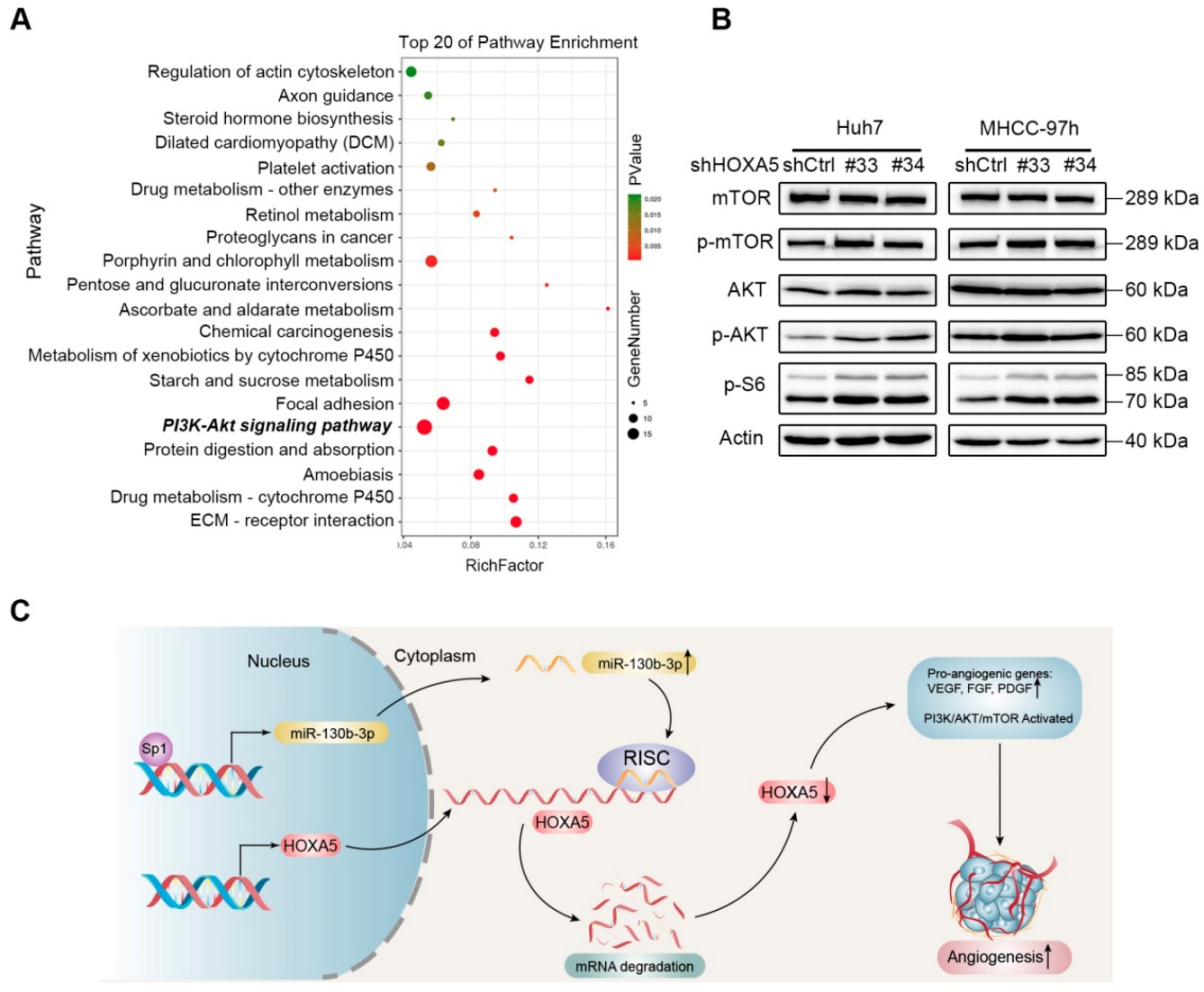
The down-regulation of HOXA5 in HCC activates the PI3K/AKT/mTOR signaling pathway. (A) Top 20 statistics of KEGG pathway enrichment based on the differentially expressed genes (DEGs) after HOXA5 knockdown in Huh7 cells. In the scatter plot, RichFactor was the ratio of DEG numbers noted in this pathway term, to all gene numbers noted in this pathway term, indicating intensiveness. P value ranges from 0 to 1, and a lower P-value represents greater intensity. The PI3K/AKT/mTOR signaling pathway was one of the most regulated biofunctions upon HOXA5 knockdown. (B) The down-regulation of HOXA5 increased the phosphorylation of the PI3K/AKT/mTOR signaling pathway-related proteins. (C) Schematic regulatory network of the Sp1/miR-130b-3p/HOXA5 axis that regulates angiogenesis in HCC.

To further explore the molecular mechanism of the anti-angiogenesis function of HOXA5, transcriptome profiling by RNA-Seq was performed. Kyoto Encyclopedia of Genes and Genomes (KEGG) pathway enrichment analysis showed that the PI3K/AKT/mTOR signaling pathway was significantly altered upon knocking-down HOXA5 (Figure 8A). Furthermore, the phosphorylation of the mammal target of rapamycin (mTOR) and other proteins, such as protein kinase B (AKT) and p70 S6 kinase, involved in the PI3K/AKT/mTOR signaling pathway were upregulated in cells transfected with shHOXA5 (Figure 8B). Additionally, HOXA5 has been reported to be involved in several pathways, including NF-kb, P53, STAT3 and MMP2. However, no positive relationship between HOXA5 and the above reported pathways were detected in this present study (Figure S8).

## Discussion

HOXA5 has been reported to be deregulated in several malignant tumors, including colorectal cancer, lung cancer, cervical cancer, and gastric cancer 31-35. However, the biological role and underlying mechanism of HOXA5 in HCC are still unclear. In the present study, we investigated the biological function and molecular mechanism of HOXA5 in HCC. Our results demonstrated that HOXA5 was down-regulated in HCC tissues and its expression was closely correlated with tumor size. Survival analyses indicated that a low HOXA5 expression predicted unfavorable OS and high recurrence probability in HCC patients. Functional experiments showed that HOXA5 significantly suppressed HCC angiogenesis.

As a member of the HOX family, HOXA5 plays an important role in tumor development and progression. Previous studies indicated that HOXA5 could up-regulated linc00312 expression, and inhibit proliferation and promote apoptosis in non-small cell lung cancer 34. HOXA5 has also been reported to be down-regulated by miR-429, and promote colorectal cancer progression and metastasis 35. Moreover, HOXA5 was observed to inhibit proliferation and induce apoptosis in cervical cancer cells via regulation of protein kinase B and p27 32. In this present study, we attempted to investigate the biological function and molecular mechanism of HOXA5 in HCC progression. Our results demonstrated that HOXA5 was down-regulated in HCC tissues and its expression closely correlated with tumor size. Survival analyses indicated that a low HOXA5 expression predicted unfavorable OS and high recurrence probability in HCC patients. It is worth noting that previous studies considered HOXA5 as a tumor-suppressor mainly due to its capacity to inhibit tumor proliferation and metastasis but not angiogenesis. The hypervascular nature underlines the importance of angiogenesis in most HCC tumors 36.Based on our research, we may be the first to discover that both the *in vitro* and *in vivo* down-regulation of HOXA5 promoted angiogenesis, and HOXA5 was regulated by the Sp1/miR-130b-3p axis and inhibited angiogenesis in HCC via the PI3K/AKT/mTOR signaling pathway.

According to the in silico target prediction algorithms, miR-130b-3p has a complementary binding sequence in the 3'-UTR of HOXA5 mRNA. Previous studies have shown that the miR-130b-3p expression was significantly upregulated in HCC and it emerged as an oncogene 37, 38. However, a recent report demonstrated that miR-130b-3p inhibited, rather than promoted, cell motility in hepatoma cell lines and may function as a tumor suppressor 39. In this current study, we found that miR-130b-3p promoted angiogenesis in HCC by directly targeting HOXA5. In addition, high miR-130b-3p expression levels were significantly associated with aggressive characteristics and poor prognosis in HCC patients. Based on the previous works from other groups and this study findings, we confirmed miR-130b-3p as an oncogene in HCC.

Sp1 is considered as a basal transcription factor and has been found to regulate angiogenesis in multiple tumors. Chen et al reported that JWA suppresses tumor angiogenesis in gastric cancer by inhibiting Sp1 activity and downregulating the proangiogenic MMP-2 expression 40. However, the specific role of Sp1 in HCC angiogenesis has not been clarified. Here, we demonstrated that Sp1 regulated HCC angiogenesis by transcriptional activation of miR-130b-3p based on the following evidences. First, the knockdown of Sp1 dramatically decreased the miR-130b-3p promoter activity and the cellular miR-130b-3p expression. Second, mutation of the potential Sp1 binding site significantly reduced the miR-130b-3p promoter activity. Third, ChIP assays revealed that Sp1 interacted with the miR-130b-3p promoter sequence *in vivo*.

The PI3K/AKT/mTOR pathway is activated in majority of human cancers and plays a key role in tumor angiogenesis 41, 42. Although anti-angiogenesis drugs such as sorafenib and regorafenib, have been approved for advanced HCC, the prognoses of these patients are still unsatisfactory. It implies that the molecular mechanism of abnormal hypervascular in HCC needs to be more intensely investigated. Although our present study indicated that the down-regulation of HOXA5 activated the PI3K/AKT/mTOR pathway, however, the detail mechanism of how HOXA5 regulates the PI3K/AKT/mTOR pathway needs to be further clarified.

## Conclusions

In summary, we demonstrated that miR-130b-3p was frequently up-regulated in HCC and was correlated with poor prognosis. HOXA5 was identified as a direct target of miR-130b-3p. Furthermore, we demonstrated that the down-regulation of HOXA5 played a crucial role in angiogenesis for HCC. In addition, miR-130b-3p was found to be transcriptionally regulated by Sp1. Our findings highlight the importance of Sp1/miR-130b-3p/HOXA5 axis in tumor angiogenesis and recurrence, and implicate HOXA5 as a potential target for HCC treatment.

## Supplementary Material

Supplementary methods, figures, and tables.

## Abbreviations

CAM: Chicken chorioallantoic membrane
ChIP: Chromatin immunoprecipitation
Ct: cycle threshold
DMEM: Dulbecco's Modified Eagle Medium
FBS: fetal bovine serum
HCC: hepatocellular carcinoma
HUVEC: Human umbilical vein endothelial cell
IHC: immunohistochemistry
miRNA: microRNA
mTOR: mammal target of rapamycin
PBS: phosphate -buffered saline
qRT-PCR: Real-time quantitative polymerase chain reaction
SFM: serum free medium
siRNA: small interference RNA
TCM: tumor cell-conditioned medium

## References

[1] Hanahan D, Weinberg RA (2011). Hallmarks of cancer: the next generation. Cell.

[2] Qian CN, Pezzella F (2018). Tumor vasculature: a sally port for inhibiting cancer cell spreading. Cancer Commun (Lond).

[3] Heimbach JK, Kulik LM, Finn RS, Sirlin CB, Abecassis MM, Roberts LR (2018). AASLD guidelines for the treatment of hepatocellular carcinoma. Hepatology.

[4] He W, Zheng Y, Zou R, Shen J, Yang J, Qiu J (2018). Long- versus short-interval follow-up after resection of hepatocellular carcinoma: a retrospective cohort study. Cancer Commun (Lond).

[5] Pan YX, Chen JC, Fang AP, Wang XH, Chen JB, Wang JC (2019). A nomogram predicting the recurrence of hepatocellular carcinoma in patients after laparoscopic hepatectomy. Cancer Commun (Lond).

[6] Welti J, Loges S, Dimmeler S, Carmeliet P (2013). Recent molecular discoveries in angiogenesis and antiangiogenic therapies in cancer. J Clin Invest.

[7] Villanueva A (2019). Hepatocellular Carcinoma. N Engl J Med.

[8] Khan KA, Kerbel RS (2018). Improving immunotherapy outcomes with anti-angiogenic treatments and vice versa. Nature Reviews Clinical Oncology.

[9] Fang L, Deng Z, Shatseva T, Yang J, Peng C, Du WW (2011). MicroRNA miR-93 promotes tumor growth and angiogenesis by targeting integrin-beta8. Oncogene.

[10] Umezu T, Tadokoro H, Azuma K, Yoshizawa S, Ohyashiki K, Ohyashiki JH (2014). Exosomal miR-135b shed from hypoxic multiple myeloma cells enhances angiogenesis by targeting factor-inhibiting HIF-1. Blood.

[11] He L, Zhu W, Chen Q, Yuan Y, Wang Y, Wang J (2019). Ovarian cancer cell-secreted exosomal miR-205 promotes metastasis by inducing angiogenesis. Theranostics.

[12] Xu Q, Liu LZ, Qian X, Chen Q, Jiang Y, Li D (2012). MiR-145 directly targets p70S6K1 in cancer cells to inhibit tumor growth and angiogenesis. Nucleic Acids Res.

[13] Sun CY, She XM, Qin Y, Chu ZB, Chen L, Ai LS (2013). miR-15a and miR-16 affect the angiogenesis of multiple myeloma by targeting VEGF. Carcinogenesis.

[14] Zhao Z, Li L, Du P, Ma L, Zhang W, Zheng L (2019). Transcriptional Downregulation of miR-4306 serves as a New Therapeutic Target for Triple Negative Breast Cancer. Theranostics.

[15] Wei R, Huang GL, Zhang MY, Li BK, Zhang HZ, Shi M (2013). Clinical significance and prognostic value of microRNA expression signatures in hepatocellular carcinoma. Clin Cancer Res.

[16] McGinnis W, Krumlauf R (1992). Homeobox genes and axial patterning. Cell.

[17] Scott MP (1992). Vertebrate homeobox gene nomenclature. Cell.

[18] Krumlauf R (1994). Hox genes in vertebrate development. Cell.

[19] Li H, Zhang Y, Chen SW, Li FJ, Zhuang SM, Wang LP (2014). Prognostic significance of Flotillin1 expression in clinically N0 tongue squamous cell cancer. Int J Clin Exp Pathol.

[20] Hou J, Zhou Y, Zheng Y, Fan J, Zhou W, Ng IO (2014). Hepatic RIG-I predicts survival and interferon-alpha therapeutic response in hepatocellular carcinoma. Cancer Cell.

[21] Tan Z, Chen K, Wu W, Zhou Y, Zhu J, Wu G (2018). Overexpression of HOXC10 promotes angiogenesis in human glioma via interaction with PRMT5 and upregulation of VEGFA expression. Theranostics.

[22] Zhuang SM, Zhang GH, Chen WK, Chen SW, Wang LP, Li H (2013). Survival study and clinicopathological evaluation of trichilemmal carcinoma. Mol Clin Oncol.

[23] Jiang L, Lin C, Song L, Wu J, Chen B, Ying Z (2012). MicroRNA-30e* promotes human glioma cell invasiveness in an orthotopic xenotransplantation model by disrupting the NF-kappaB/IkappaBalpha negative feedback loop. J Clin Invest.

[24] Cai MY, Tong ZT, Zheng F, Liao YJ, Wang Y, Rao HL (2011). EZH2 protein: a promising immunomarker for the detection of hepatocellular carcinomas in liver needle biopsies. Gut.

[25] Lin XJ, Fang JH, Yang XJ, Zhang C, Yuan Y, Zheng L (2018). Hepatocellular Carcinoma Cell-Secreted Exosomal MicroRNA-210 Promotes Angiogenesis In Vitro and In Vivo. Mol Ther Nucleic Acids.

[26] Li JH, Liu S, Zhou H, Qu LH, Yang JH (2014). starBase v2.0: decoding miRNA-ceRNA, miRNA-ncRNA and protein-RNA interaction networks from large-scale CLIP-Seq data. Nucleic Acids Res.

[27] Yeung ML, Yasunaga J, Bennasser Y, Dusetti N, Harris D, Ahmad N (2008). Roles for microRNAs, miR-93 and miR-130b, and tumor protein 53-induced nuclear protein 1 tumor suppressor in cell growth dysregulation by human T-cell lymphotrophic virus 1. Cancer Res.

[28] Liu N, Ding D, Hao W, Yang F, Wu X, Wang M (2016). hTERT promotes tumor angiogenesis by activating VEGF via interactions with the Sp1 transcription factor. Nucleic Acids Res.

[29] Chen Y, Huang Y, Huang Y, Xia X, Zhang J, Zhou Y (2014). JWA suppresses tumor angiogenesis via Sp1-activated matrix metalloproteinase-2 and its prognostic significance in human gastric cancer. Carcinogenesis.

[30] Cho SG, Yi Z, Pang X, Yi T, Wang Y, Luo J (2009). Kisspeptin-10, a KISS1-derived decapeptide, inhibits tumor angiogenesis by suppressing Sp1-mediated VEGF expression and FAK/Rho GTPase activation. Cancer Res.

[31] Hu LL, Wang XX, Chen X, Chang J, Li C, Zhang Y (2007). BCRP gene polymorphisms are associated with susceptibility and survival of diffuse large B-cell lymphoma. Carcinogenesis.

[32] Cheng J, Huo DH, Kuang DM, Yang J, Zheng L, Zhuang SM (2007). Human macrophages promote the motility and invasiveness of osteopontin-knockdown tumor cells. Cancer Res.

[33] Xu T, Zhu Y, Xiong Y, Ge YY, Yun JP, Zhuang SM (2009). MicroRNA-195 suppresses tumorigenicity and regulates G1/S transition of human hepatocellular carcinoma cells. Hepatology.

[34] Zhu Q, Lv T, Wu Y, Shi X, Liu H, Song Y (2017). Long non-coding RNA 00312 regulated by HOXA5 inhibits tumour proliferation and promotes apoptosis in Non-small cell lung cancer. J Cell Mol Med.

[35] Han Y, Zhao Q, Zhou J, Shi R (2017). miR-429 mediates tumor growth and metastasis in colorectal cancer. Am J Cancer Res.

[36] Morse MA, Sun W, Kim R, He AR, Abada PB, Mynderse M (2019). The Role of Angiogenesis in Hepatocellular Carcinoma. Clin Cancer Res.

[37] Ma S, Tang KH, Chan YP, Lee TK, Kwan PS, Castilho A (2010). miR-130b Promotes CD133(+) liver tumor-initiating cell growth and self-renewal via tumor protein 53-induced nuclear protein 1. Cell Stem Cell.

[38] Tu K, Zheng X, Dou C, Li C, Yang W, Yao Y (2014). MicroRNA-130b promotes cell aggressiveness by inhibiting peroxisome proliferator-activated receptor gamma in human hepatocellular carcinoma. Int J Mol Sci.

[39] Lin YH, Wu MH, Liao CJ, Huang YH, Chi HC, Wu SM (2015). Repression of microRNA-130b by thyroid hormone enhances cell motility. J Hepatol.

[40] Liu JJ, Lin XJ, Yang XJ, Zhou L, He S, Zhuang SM (2014). A novel AP-1/miR-101 regulatory feedback loop and its implication in the migration and invasion of hepatoma cells. Nucleic Acids Res.

[41] Karar J, Maity A (2011). PI3K/AKT/mTOR pathway in angiogenesis. Frontiers in molecular neuroscience.

[42] Zhao P, Chen H, Wen D, Mou S, Zhang F, Zheng S (2018). Personalized treatment based on mini patient-derived xenografts and WES/RNA sequencing in a patient with metastatic duodenal adenocarcinoma. Cancer Commun (Lond).

