# Supplementary material for: Dysregulated Sp1/miR-130b-3p/HOXA5 axis contributes to tumor angiogenesis and progression of hepatocellular carcinoma
Source: Theranostics. 2020 Apr 6;10(12):5209–24. doi: 10.7150/thno.43640 (PMC7196310; doi:10.7150/thno.43640)
Supplement: Supplementary file 1 — Supplementary methods, figures, and tables. [file thnov10p5209s1.pdf]

Dysregulated Sp1/miR-130b-3p/HOXA5 axis contributes to tumor angiogenesis and progression of hepatocellular carcinoma

Yadi Liao[1*], Chenwei Wang[1,2*], Zhiwen Yang[1,2], Wenwu Liu[1], Yichuan Yuan[1,2], Kai Li[1,2], Yuanping Zhang[1,2], Yongjin Wang[1,2],Yunxing Shi[1,2], Yuxiong Qiu[1,2], Dinglan Zuo[1], Wei He[1,2],Jiliang Qiu[1,2], Xinyuan Guan[1,3], Yunfei Yuan[1,2✉], Binkui Li[1,2✉]

Inventory of supplementary data

1. Supplementary Methods…………………………………..Page 2 - 4

2. Supplementary Figures and Legends………………..…..Page 5 - 16

3. Supplementary Tables……………..………..…..……….Page 17 - 28

Supplementary Methods

Cell lines and human umbilical vein endothelial cells.

Human HCC cell lines (Huh7, BEL-7402, SMMC-7721, L02, HepG2, SK-hep1, MHCC-LM3, QGY-7703, PLC/PRF/5, Hep3B, MHCC-97H, and MHCC-97L), and transformed human embryonic kidney (HEK293T) cell line were maintained in Dulbecco's modified Eagles's medium (DMEM, Invitrogen, NY) supplemented with 10% fetal bovine serum (FBS, Hyclone, Logan, UT). The Huh7 and BEL-7402 cell sublines, which stably expressed miR-130b-3p (Huh7-miR-130b-3p, and BEL-7402-miR-130b-3p), and the matched control lines (Huh7-vector, and BEL-7402-vector) were established with the Lenti-PacTM HIV Expression Packaging System (GeneCopoeia, Rockville, MD, USA). Similarly, the Huh7 and MHCC-97H cell sublines, which stably knockdown HOXA5 (Huh7-shHOXA5 #33 and #34, MHCC-97H-shHOXA5#33 and #34), and the matched control lines (Huh7-shCtrl, and MHCC-97H-shCtrl) were established.

Human umbilical vein endothelial cells (HUVECs) were isolated and maintained in serum-free medium for endothelial cells (SFM, Invitrogen). The primary HUVECs were used at passages 3-6 in all experiments.

RNA oligoribonucleotides and vectors.

All miRNA mimic and small interference RNA (siRNA) duplexes (Table S5) were purchased from Genepharma (Shanghai, P.R. China). Si-HOXA5 targeted mRNA of human HOXA5 (GenBank accession no. NM_019102.3). Si-Sp1 targeted mRNA of human Sp1 (GenBank accession no. NM_138473). The negative

control RNA duplex (NC) for both miRNA mimic and siRNA was nonhomologous to any human genome sequence.

miR-130b-3p was overexpressed using pEZX-MR03, while miR-130b-3p inhibitor was overexpressed using pEZX-AM03 vector. HOXA5 was overexpressed using pEZ-Lv105, while HOXA5 was knockdown using psi-LVRU6GP vector. Sp1 was overexpressed using pEZ-Lv105. Wildtype (WT) and mutant (MUT) HOXA5-3'UTR were inserted into pEZX-MT01 firefly luciferase reporter plasmid.

To construct the firefly luciferase reporter plasmids for verifying the miR-130b-3p promoter region, the genomic fragments upstream of mature miR-130b-3p were cloned into the EcoRI and HindIII sites upstream of the firefly luciferase gene in pEZX-PG04.1. The plasmid with deletion of the potential binding site of Sp1 was generated by fusion PCR based on the wild-type construct. All of the vectors were purchased from Genecopoeia (Guangzhou, China).

Esablishemnt of stable knockdown and overexpression HCC cells.

Human miR-130b-3p and HOXA5 knockdown or overexpression HCC cells were generated by lentiviral mediated approach. HEK293T cells (1.5×10^6) were plated in a 10 cm dish and maintained in DMEM with 10% FBS.After 48 hours, HEK293T cells were transfected with indicated lentiviral vectors, using Lenti-PacTM HIV Expression Packaging Kit according to the manufacturer's instructions. Collect the pseudovirus-containing culture medium in sterile tubes 48 h post transfection and centrifuge the tubes at 500 g for 10 min to get rid of cell debris. Following centrifugation, the supernatant was passed through a 0.45

μm filter and added to the target cells (Huh7 and BEL-7402 cells) in the presence of 5 μg/mL Polybrene. Puromycin (final concentration: 1.5 μg/mL) or Hygromycin (100 μg/mL) was used to select stable clones.

Cell transfection.

Reverse transfection of RNA oligoribonucleotides were performed with Lipofectamine-RNAiMAX (Invitrogen). Fifty nM of RNA duplex and 100 nM of inhibitor were used for each transfection. Cotransfection of RNA duplex and plasmid DNA was performed with Lipofectamine 2000 (Invitrogen). Cell transfection was performed according to the manufacturer's instructions (Invitrogen, Carlsbad, CA).

Supplementary Figures and Legends

Figure S1

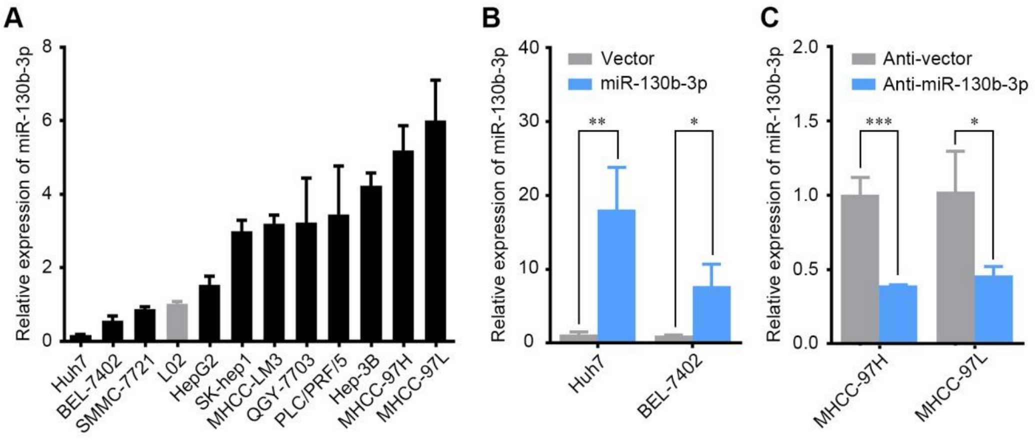

Figure S1. The expression of miR-130b-3p in HCC wild type and stable cell lines.

(A) The expression of miR-130b-3p in HCC wild type cell lines.

(B) The up-regulated expression of miR-130b-3p was confirmed by qRT-PCR.

(C) The down-regulated expression of miR-130b-3p was confirmed by qRT-PCR.

Figure S2

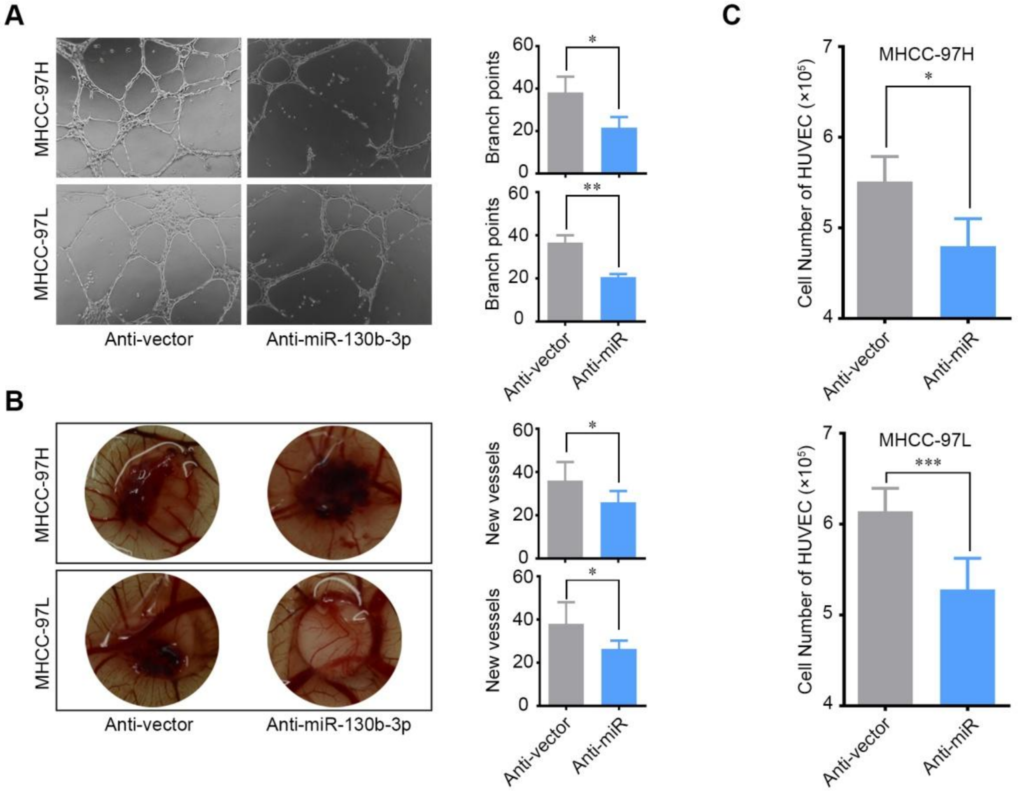

Figure S2. The down-regulation of miR-130b-3p inhibits tumor angiogenesis in vitro.

(A) The down-regulation of miR-130b-3p inhibited tube formation of HUVECs. HUVECs were cultured in TCM from the indicated cells. Representative images of capillary-like structures and the number of branch points of HUVECs are presented.

(B) Effect of miR-130b-3p on vascularization in the CAM angiogenesis model. Filter discs soaked with TCM were loaded on the CAMs of day-8 chick embryos. After 5 days incubation, the area under and surround the filter was fixed and

photographed. Representative images of neovascularization and the number of new blood vessels are presented.

(C) The down-regulation of miR-130b-3p inhibits HUVECs proliferation *in vitro*. HUVEC cells were seeded on the 6-well plate with a density of 3×10^5 per well, and cultured with SFM supplemented with 20% FBS and 0.3% EGF for 6 h. Then the above medium was replaced with TCM from indicated cells and cultured for additional 24 h. The numbers of HUVEC cells were counted using the ScepterTM Handheld Automated Cell Counter. Results were based on 6 independent experiments.

Figure S3

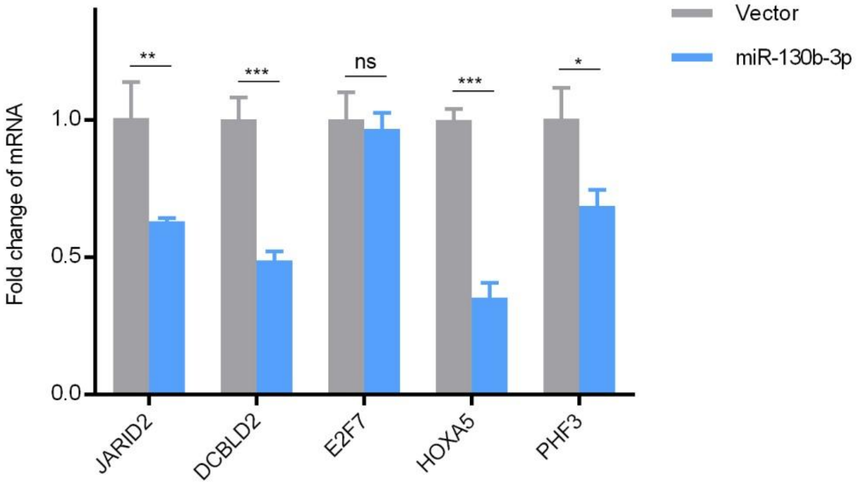

Figure S3. PCR screen for potential targets of miR-130b-3p.

The top 5 potential targets of miR-130b-3p, which were predicted by bioinformatics algorithms, were screened by qRT-PCR. Among them, HOXA5 was shown to be the most significantly downregulated target in HCC cells transfected with miR-130b-3p.

106 **Figure S4**

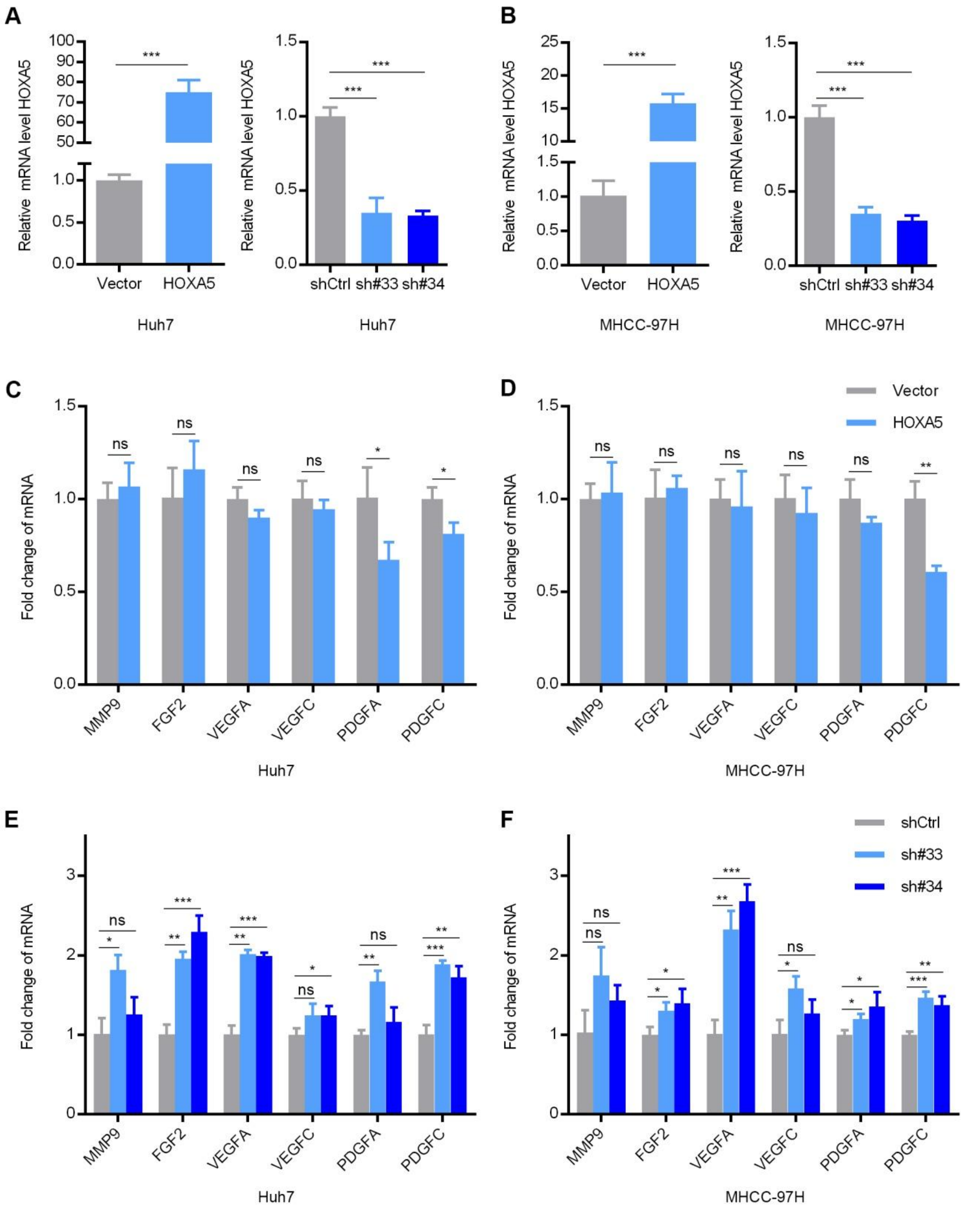

107

Figure S4. The mRNA level of angiogenesis relevant genes was upregulated by HOXA5.

(A) The up-regulation of HOXA5 (left) and knockdown efficiency of shHOXA5 (right) in Huh7 cells were confirmed by qRT-PCR.

(B) The up-regulation of HOXA5 (left) and knockdown efficiency of shHOXA5 (right) in MHCC-97H cells were confirmed by qRT-PCR.

(C) (D) The mRNA level of angiogenesis relevant genes was regulated by ectopic expression of HOXA5. The expression of MMP9, FGF2, VEGFA, VEGFC, PDGFA, and PDGFC in Huh7 (C) or MHCC-97H (D) cells transfected with HOXA5 or vector was determined by qRT-PCR.

(E) (F) The down-regulation of HOXA5 increased the mRNA level of angiogenesis relevant genes. The expression of MMP9, FGF2, VEGFA, VEGFC, PDGFA, PDGFC in Huh7 (E) or MHCC-97H (F) cells transfected with shHOXA5 or control vector were determined by qRT-PCR. Results were based on at least three independent experiments.Data are presented as their mean ± SD.

ns, not significant; * $p < 0.05$; ** $p < 0.01$; *** $p < 0.001$.

Figure S5

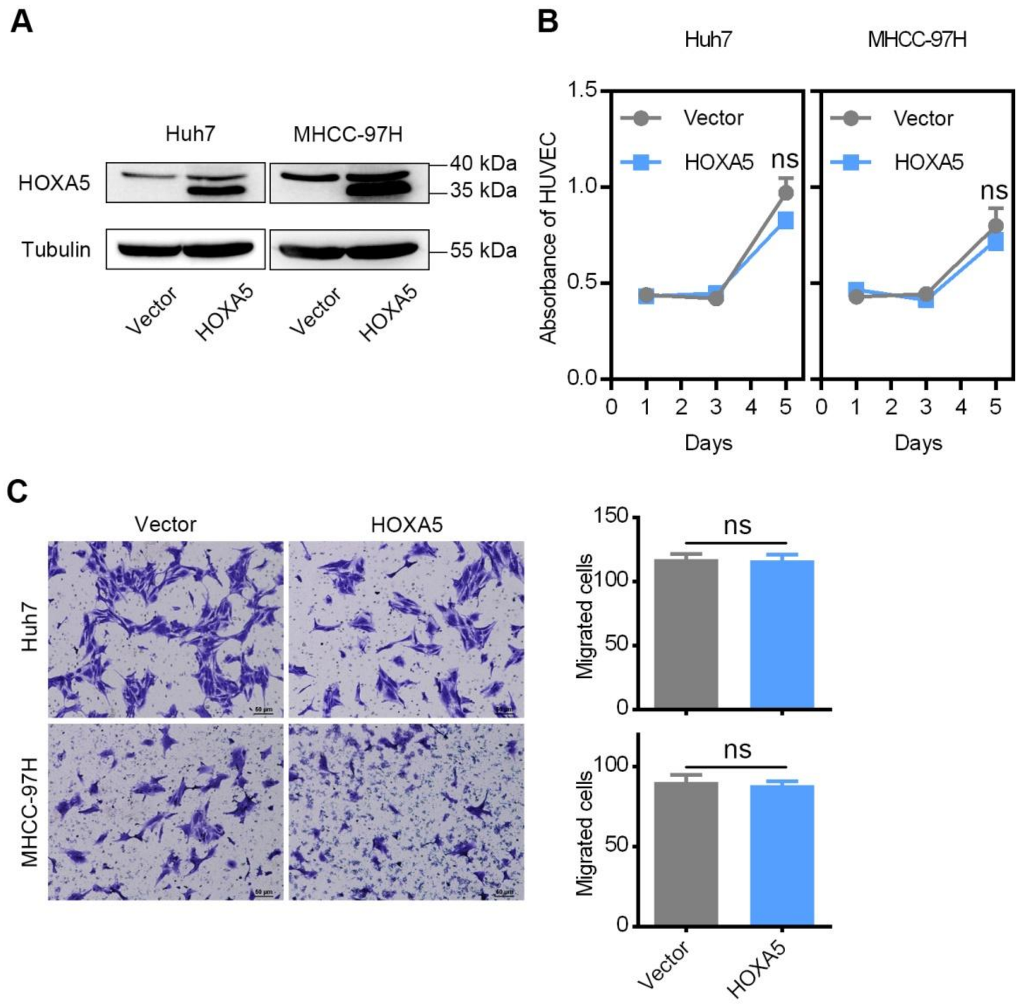

Figure S5. Ectopic expression of HOXA5 did not alter the angiogenesis capacity in HCC.

(A) Western blot analysis showing ectopic expression of HOXA5 in transfected Huh7 and MHCC-97H cells.

(B) The TCM from HOXA5 overexpressed HCC cells did not alter the proliferation of HUVECs. HUVECs were grown in complete medium for 12 h at 37° C in a 96-

well plate and then replaced with TCM and cultured for indicated hours. Cell viability was measured by CCK-8 assay. Three independent experiments were performed.

(C) The TCM from HOXA5 overexpressed HCC cells did not alter the migration of HUVECs. HUVECs were seeded in the upper transwell chambers with the TCM in the lower compartments and incubated for 12 h.

* $p < 0.05$; ** $p < 0.01$.

Figure S6

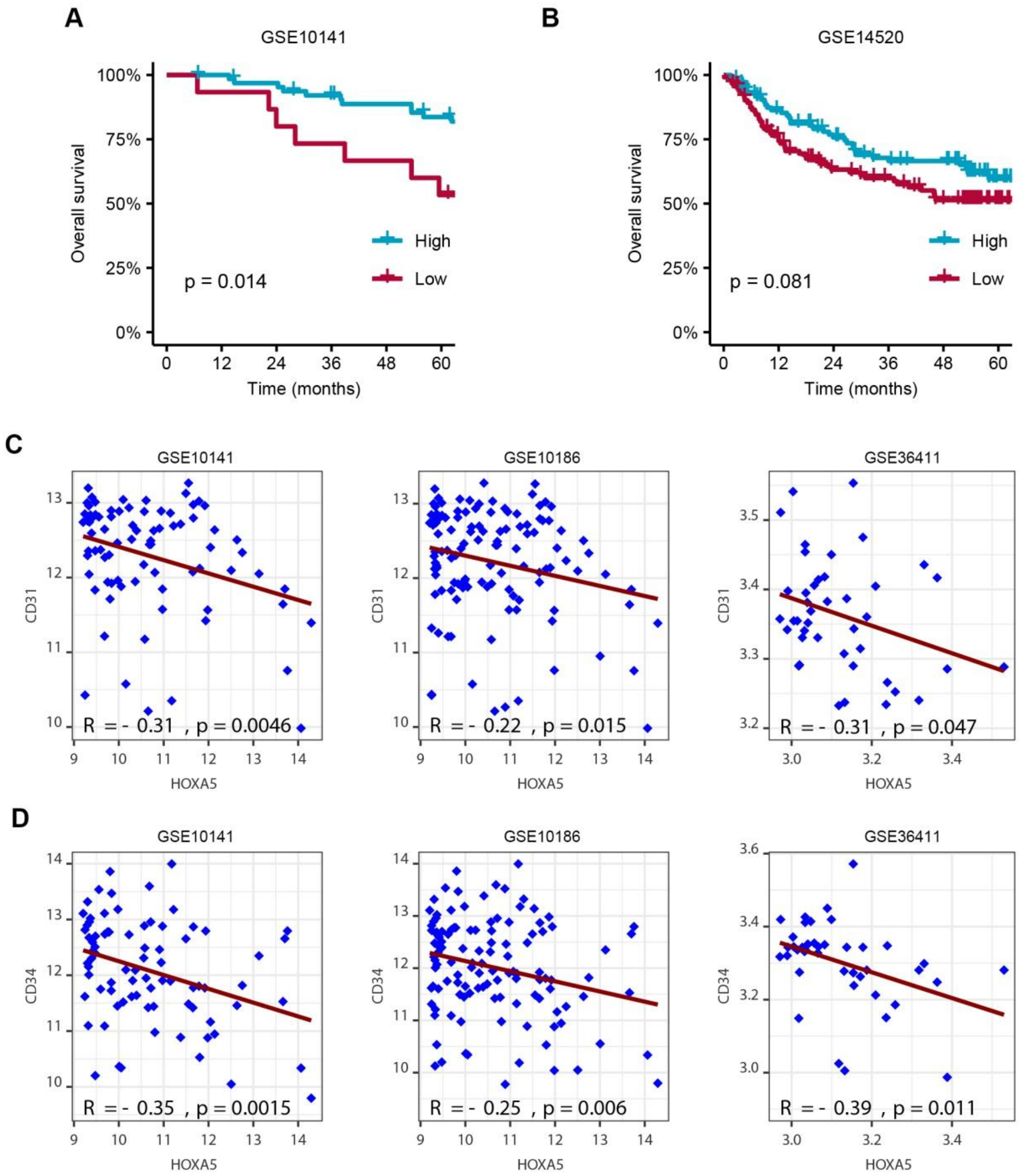

Figure S6. Decreased expression of HOXA5 associated with poor prognosis and angiogenesis in HCC patients.

(A) Overall survival of the public dataset of 80 HCC cases (GSE10141) based on HOXA5 expression.

(B) Overall survival of the public dataset of 221 HCC cases (GSE14520) based on HOXA5 expression.

(C) Scatter-plots showing correlation of HOXA5 and CD31 in HCC patients from the GEO database (GSE10141, n = 80; GSE10186, n = 118; GSE36411, n = 42).

(D) Scatter-plots showing correlation of HOXA5 and CD34 in HCC patients from the GEO database (GSE10141, n = 80; GSE10186, n = 118; GSE36411, n = 42).

Figure S7

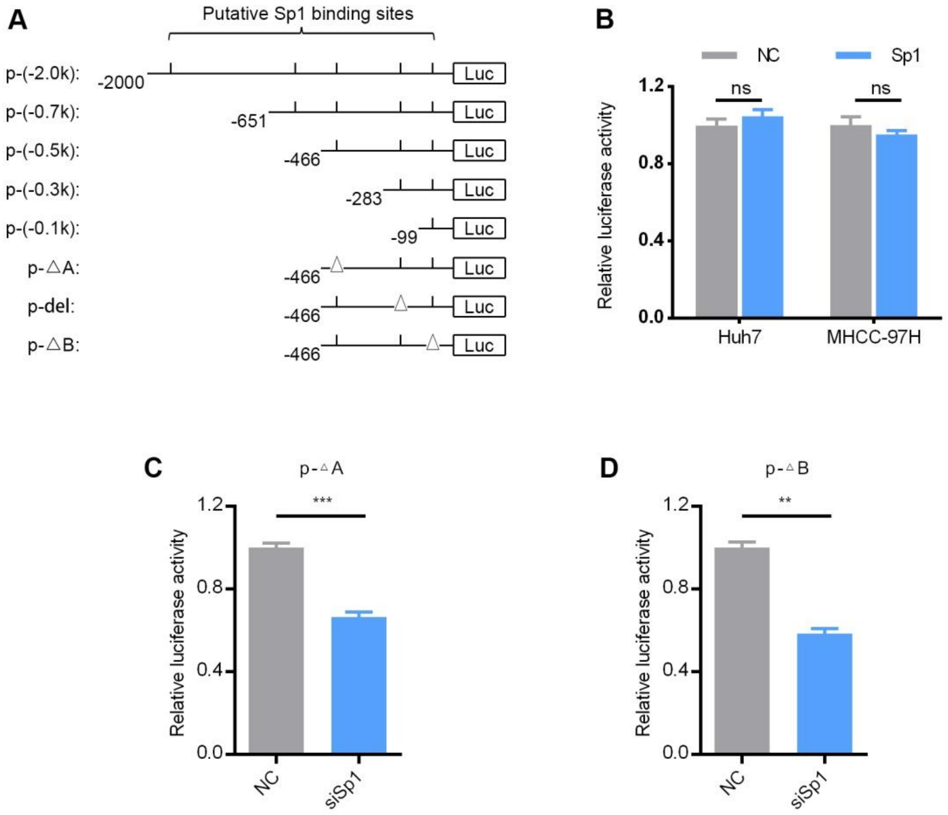

Figure S7. Regulation of miR-130b-3p by Sp1 in HCC cells.

(A) Schematic diagram of firefly luciferase reporter constructs containing the indicated genomic fragments upstream of miR-130b-3p gene. Putative Sp1 binding sites are depicted as short vertical lines. Deletion of the Sp1 binding site is depicted as triangle (Δ).

(B) Ectopic expression of Sp1 did not change the promoter activity of p-(-2.0 k).

(C) Silencing of Sp1 expression reduced the promoter activity of p-(ΔA).

(D) Silencing of Sp1 expression reduced the promoter activity of p-(ΔB).

Figure S8

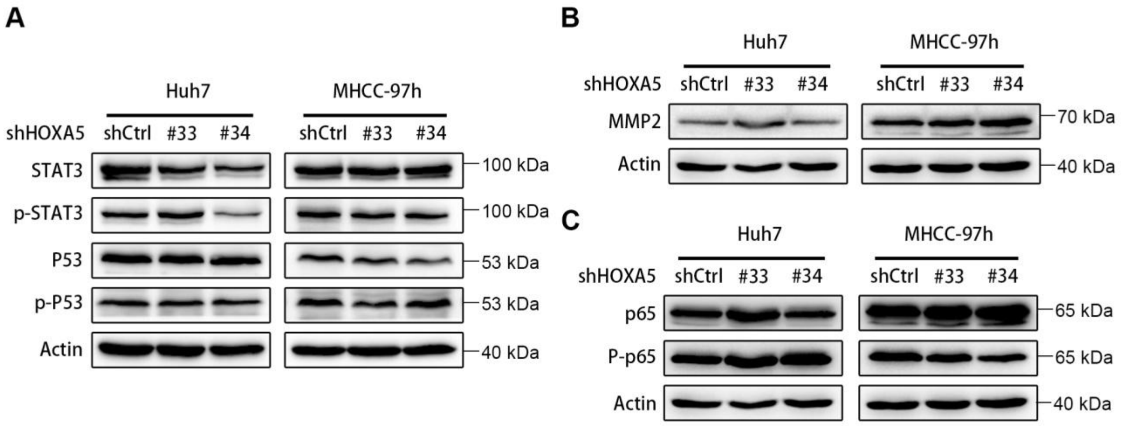

Figure S8. Effect of HOXA5 Silencing on signaling pathways.

(A) HOXA5 Silencing did not affect phosphorylation of STAT3 and P53 in HCC cells.

(B) HOXA5 silencing did not affect expression of MMP2 in HCC cells.

(C) HOXA5 silencing did not affect phosphorylation of P65 in HCC cells.

Table S1. Association of miR-130b-3p with clinical features in cohort 1.

		miR-130b-3p		
		**High (n = 53)**	**Low (n = 54)**	**P value[a]**
Gender	M vs. F	50 (94.3%)/3 (5.7%)	47 (87.0%)/7 (13.0%)	0.320
Age	>50 vs. ≤50 yrs	23 (43.4%)/30 (56.6%)	33 (61.1%)/21	0.067
HBV	+ vs. -[b]	45 (84.9%)/8 (15.1%)	42 (77.8%)/12	0.344
AFP	>200 vs. ≤200 ng/mL	22 (41.5%)/31 (58.5%)	31 (57.4%)/ 23	0.100
Cirrhosis	+ vs. -	40 (75.5%)/13 (24.5%)	42 (75.5%)/12	0.778
Tumor size	>7 vs. ≤7 cm	33 (62.3%)/20 (37.7%)	23 (42.6%)/31	**0.042**
Tumor number	>1 vs. 1	19 (35.8%)/34 (64.2%)	18 (33.3%)/36	0.784
MVI[c]	+ vs. -	15 (28.3%)/38 (71.7%)	9 (16.7%)/45 (83.3%)	0.149
Edmondson grade	III-IV vs. I-II	21 (39.6%)/32 (60.4%)	24 (44.4%)/30	0.613
TNM stage	>I vs. I	32 (60.4%)/21 (39.6%)	24 (44.4%)/30	0.099

[a]*P* values were calculated using Chi-squared test.

[b] +, presence; -, absence.

[c] MVI, microscopic vascular invasion.

Table S2. Univariate and multivariate analysis of factors associated with overall and recurrence-free survival in cohort 1.

Characteristic	Case Number	Overall survival		Recurrence-free survival	
		HR(95% CI)[a]	P	HR(95% CI)	P
**Univariate analysis**					
miR-130b-3p (High vs. Low)[b]		1.936 (1.030-3.637)	**0.040**	2.356 (1.399-3.965)	**0.001**
Gender (M vs. F)		1.150 (0.355-3.730)	0.816	1.254 (0.502-3.132)	0.628
Age (>50 vs. ≤50 yrs)		1.302 (0.695-2.439)	0.410	0.755 (0.457-1.248)	0.273
HBV (+ vs. -)[c]		2.203 (0.861-5.638)	0.099	2.025 (0.962-4.265)	0.063
AFP (>200 vs. ≤200 ng/mL)		1.984 (1.058-3.720)	**0.033**	1.840 (1.108-3.055)	**0.019**
Cirrhosis (+ vs. -)		0.924 (0.654-1.306)	0.656	0.888 (0.496-1.591)	0.689
Tumor size (>7 vs. ≤7 cm)		3.269 (1.663-6.425)	**0.001**	3.127 (1.822-5.368)	**< 0.001**
Tumor number (>1 vs. 1)		1.562 (0.843-2.896)	0.157	1.774 (1.067-2.950)	**0.027**
MVI (+ vs. -)		3.311 (1.761-6.226)	**< 0.001**	2.935 (1.694-5.086)	**< 0.001**
Edmondson grade (III-IV vs. I-II)		1.863 (1.008-3.445)	**0.047**	1.770 (1.071-2.927)	**0.026**

TNM stage (>I vs. I)	2.534 (1.291-4.973)	**0.007**	2.537 (1.491-4.317)	**0.001**
**Multivariate analysis**				
miR-130b-3p (High vs. Low)	2.076 (1.009-4.274)	**0.047**	3.203 (1.707-6.009)	**< 0.001**
AFP (>200 vs. ≤200 ng/mL)	-	**-**	2.125 (1.169-3.863)	**0.013**
Tumor size (>7 vs. ≤7 cm)	-	-	2.128 (1.170-3.871)	**0.013**
Edmondson grade (III-IV vs. I-II)	-	-	1.848 (1.080-3.165)	**0.025**

[a]HR (hazard ratio) and P values were calculated using univariate or multivariate Cox proportional hazards regression; 95% CI, 95% confidence interval.

[b]miR-130b-3p level was examined in 107 HCC tissues by qPCR and normalized to U6 level. The 50th percentile value of the examined samples was chosen as the cut-off point to separate miR-130b-3p-low from miR-130b-3p-high expression groups.

[c]+, presence; -, absence.

Table S3. Association of HOXA5 with clinical features in cohort 2.

		HOXA5		
		**High (n = 229)**	**Low (n = 220)**	**P value[a]**
Gender	M vs. F	203(88.6%)/26(11.4%)	202(91.8%)/18(8.2%)	0.331
Age	>50 vs. ≤50 yrs	133(58.1%)/96(41.9%)	114(51.8%)/106(48.2%)	0.216
HBV	+ vs. -[b]	205(89.5%)/24(10.5%)	199(90.5%)/21(9.5%)	0.863
AFP	>400 vs. ≤400 ng/mL	73(31.9%)/156(68.1%)	93(42.3%)/127(57.7%)	**0.029**
Cirrhosis	+ vs. -	176(76.9%)/53(23.1%)	180(81.8%)/40(18.2%)	0.238
Tumor size	>5 vs. ≤5 cm	116(50.7%)/113(49.3%)	144(65.5%)/76(34.5%)	**0.002**
Tumor number	>1 vs. 1	45(19.7%)/184(80.3%)	46(21.0%)/174(79.0%)	0.830
MVI[c]	+ vs. -	80(34.9%)/149(65.1%)	62(28.2%)/158(71.8%)	0.151
Edmondson grade	III-IV vs. I-II	84(36.7%)/145(63.3%)	99(45.0%)/121(55.0%)	0.090
TNM stage	>I vs. I	115(50.2%)/114(49.8%)	120(54.5%)/100(45.5%)	0.410

[a]*P* values were calculated using Chi-squared test.

[b] +, presence; -, absence.

[c] MVI, microscopic vascular invasion.

Table S4. Univariate and multivariate analysis of factors associated with overall and recurrence-free survival in cohort 2.

Characteristic	Case Number	Overall survival		Recurrence-free survival	
		HR(95% CI)[a]	P	HR(95% CI)	P
**Univariate analysis**					
HOXA5 (Low vs. High)[b]	229/220	1.933 (1.439-2.596)	**<0.001**	1.689 (1.299-2.197)	**<0.001**
Gender (M vs. F)	405/44	1.794 (1.000-3.220)	0.050	1.543 (0.954-2.497)	0.077
Age (>50 vs. ≤50 yrs)	247/202	0.890 (0.669-1.184)	0.423	0.707 (0.547-0.913)	**0.008**
HBV (+ vs. -)[c]	404/45	1.411 (0.833-2.391)	0.201	2.417 (1.381-4.231)	**0.002**
AFP (>400 vs. ≤400 ng/mL)	166/283	1.486 (1.116-1.980)	**0.007**	1.207 (0.927-1.572)	0.162
Cirrhosis (+ vs. -)	356/93	1.072 (0.751-1.529)	0.703	1.267 (0.910-1.764)	0.161
Tumor size (>5 vs. ≤5 cm)	260/189	1.726 (1.277-2.333)	**<0.001**	1.349 (1.039-1.751)	**0.025**
Tumor number (>1 vs. 1)	91/358	1.886 (1.372-2.593)	**<0.001**	1.632 (1.207-2.206)	**0.001**
MVI (+ vs. -)	142/307	1.458 (1.085-1.958)	**0.012**	1.138 (0.865-1.498)	0.355
Edmondson grade (III-IV vs. I-II)	183/266	1.468 (1.103-1.953)	**0.008**	1.201 (0.926-1.558)	0.168

TNM stage (>I vs. I)	235/214	1.936 (1.441-2.602)	**<0.001**	1.398 (1.080-1.809)	**0.011**
**Multivariate analysis**					
HOXA5 (Low vs. High)	229/220	1.758 (1.295-2.386)	**<0.001**	1.625 (1.239-2.132)	**<0.001**
Tumor size (>5 vs. ≤5 cm)	260/189	1.474 (1.077-2.017)	**0.015**	-	-
Tumor number (>1 vs. 1)	91/358	-	-	1.532 (1.024-2.293)	**0.038**

[a]HR (hazard ratio) and P values were calculated using univariate or multivariate Cox proportional hazards regression; 95% CI, 95% confidence interval.

[b]HOXA5 level was examined in 449 HCC tissues by immunohistochemistry The 47th percentile value of the examined samples was chosen as the cut-off point to separate HOXA5 -low from HOXA5-high expression groups.

[c]+, presence; -, absence.

Table S5. Sequences of RNA Oligonucleotides

Name	Sense Strand/Sense Primer (5'-3')	Antisense Strand/Antisense Primer (5'-3')
**miRNA and siRNA Duplexes**		
miR-130b-3p	CAGUGCAAUGAUGAAAGGGCAU	GCCCUUUCAUCAUUGCACUGUU
si-Sp1	AATGAGAACAGCAACAACTCC	GGAGTTGTTGCTGTTCTCATT
NC	UUCUCCGAACGUGUCACGUTT	ACGUGACACGUUCGGAGAATT
shHOXA5 #33	GGATTGAAATAGCACATGCTC	-
shHOXA5 #34	GCTATAGACGCACAAACGACC	-

Table S6. Potential targets of miR-130b-3p predicted by five bioinformatics algorithms,

name	geneName	position	targetScan	picTar	RNA22	PITA	miRanda
hsa-miR-130b-3p	JARID2	chr6:15521916-	2519[7]	2519[7]	2532[9]	2519[7]	2532[9]
hsa-miR-130b-3p	DCBLD2	chr3:98515494-	290[8]	290[8]	290[8]	290[8]	290[8]
hsa-miR-130b-3p	E2F7	chr12:77415244-	337[11]	337[11]	1022[12]	337[11]	2043[12]
hsa-miR-130b-3p	HOXA5	chr7:27181092-	299[14]	299[14]	299[14]	299[14]	302[15]
hsa-miR-130b-3p	PHF3	chr6:64424016-	0[2]	0[2]	0[2]	0[2]	0[2]
hsa-miR-130b-3p	HECW2	chr2:197065029-	28[1]	28[1]	28[1]	28[1]	28[1]
hsa-miR-130b-3p	BAHD1	chr15:40759300-	26[3]	26[3]	26[3]	26[3]	26[3]
hsa-miR-130b-3p	SNX27	chr1:151667243-	113[7]	113[7]	113[7]	113[7]	113[7]
hsa-miR-130b-3p	SPTY2D1	chr11:18630367-	166[9]	166[9]	212[12]	166[9]	166[9]
hsa-miR-130b-3p	SOCS5	chr2:46988493-	7[1]	7[1]	7[1]	7[1]	7[1]
hsa-miR-130b-3p	MIER1	chr1:67452939-	62[5]	31[5]	93[8]	31[5]	186[8]
hsa-miR-130b-3p	MIER1	chr1:67453270-	42[4]	42[4]	46[4]	42[4]	92[4]
hsa-miR-130b-3p	RALBP1	chr18:9537237-	8[1]	8[1]	8[1]	8[1]	8[1]
hsa-miR-130b-3p	MLL	chr11:118395373-	153[12]	153[12]	153[12]	153[12]	153[12]
hsa-miR-130b-3p	PRKD3	chr2:37478132-	21[2]	21[2]	29[3]	21[2]	29[3]

hsa-miR-130b-3p	ARHGAP12	chr10:32096474-	678[13]	678[13]	696[14]	678[13]	686[13]
hsa-miR-130b-3p	EFNB2	chr13:107142397-	160[6]	160[6]	160[6]	160[6]	160[6]
hsa-miR-130b-3p	AKAP1	chr17:55197715-	61[4]	61[4]	61[4]	61[4]	61[4]
hsa-miR-130b-3p	PPARG	chr3:12475686-	67[4]	67[4]	67[4]	67[4]	134[4]
hsa-miR-130b-3p	NPTX1	chr17:78441873-	230[12]	230[12]	230[12]	230[12]	230[12]
hsa-miR-130b-3p	MED12L	chr3:151150626-	0[6]	0[6]	0[6]	0[6]	0[6]
hsa-miR-130b-3p	RAB34	chr17:27041572-	727[13]	364[13]	364[13]	364[13]	364[13]
hsa-miR-130b-3p	ARHGEF12	chr11:120356938-	114[4]	114[4]	114[4]	114[4]	114[4]
hsa-miR-130b-3p	ZFYVE26	chr14:68213503-	5168[23]	5168[23]	5168[23]	5168[23]	5168[23]
hsa-miR-130b-3p	TNRC6A	chr16:24835520-	1180[18]	1180[18]	1195[18]	1180[18]	1195[18]
hsa-miR-130b-3p	MAP3K9	chr14:71196983-	492[15]	492[15]	492[15]	492[15]	492[15]
hsa-miR-130b-3p	RNF38	chr9:36339618-	88[5]	88[5]	88[5]	88[5]	88[5]
hsa-miR-130b-3p	SNPH	chr20:1287720-	2[3]	2[3]	2[3]	2[3]	2[3]
hsa-miR-130b-3p	OTUD3	chr1:20239075-	609[12]	609[12]	609[12]	609[12]	609[12]
hsa-miR-130b-3p	USP33	chr1:78162181-	544[9]	544[9]	549[9]	544[9]	1098[9]
hsa-miR-130b-3p	BTBD3	chr20:11906994-	6239[24]	6239[24]	6239[24]	6239[24]	6239[24]

Table S7. Potential binding sites in the promoter of miR-130b-3p predicted by AliBaba 2.1.

Transcription factor	Sites (n)	Percentage (%)
Sp1	97	41.81
C/EBPalpha	11	4.74
NF-1	10	4.31
AP-2alphaA	9	3.88
AP-2	7	3.02
NF-kappaB	7	3.02
AP-1	6	2.59
ETF	6	2.59
Krox-20	4	1.72
Oct-1	3	1.29
repressor_of_CA	3	1.29
USF	3	1.29
C/EBP	2	0.86
c-Jun	2	0.86
CPE_binding_pro	2	0.86
c-Rel	2	0.86
CTF	2	0.86
ER	2	0.86
GATA-1	2	0.86
HSTF	2	0.86
MIG1	2	0.86
NF-E2	2	0.86
NF-kappaB-like	2	0.86
Oct-1A	2	0.86
RAP1	2	0.86
TEC1	2	0.86
AP-4	1	0.43
ARP-1	1	0.43
C/EBPalpha(p20)	1	0.43
C/EBPbeta	1	0.43
C/EBPdelta	1	0.43

CACCC	1	0.43
CeMyoD	1	0.43
c-Myc	1	0.43
COUP	1	0.43
CPC1	1	0.43
CREB	1	0.43
CREMdeltaC-G	1	0.43
Da	1	0.43
delta_factor	1	0.43
Dl	1	0.43
E1	1	0.43
EFI	1	0.43
GABP	1	0.43
GCN4	1	0.43
GLI3	1	0.43
GR	1	0.43
HNF-3	1	0.43
Max1	1	0.43
MBP-1	1	0.43
MyoD	1	0.43
NF-kappaB2	1	0.43
Odd	1	0.43
Olf-1	1	0.43
Pit-1a	1	0.43
PR	1	0.43
REB1	1	0.43
RelA	1	0.43
REV-ErbAalpha	1	0.43
RXR-beta	1	0.43
SRF	1	0.43
Tra-1	1	0.43
YY1	1	0.43

Table S8. JASPAR reports for predicted TF binding sites in the putative promoter regions (-2kb to +1) of hsa-miR-130b.

Model ID	Model name	Score	Relative score	Start	End	Strand	predicted site sequence
MA0079.3	SP1	15.4315	0.975286119383	1828	1838	+	tcccctccccc
MA0079.3	SP1	14.5098	0.963690996046	1932	1942	+	gccccgcccca
MA0079.3	SP1	14.3493	0.961671715676	1421	1431	+	ccccctcccct
MA0079.3	SP1	13.0804	0.945707226065	1607	1617	+	gccccacccac
MA0079.3	SP1	12.3673	0.93673601417	1823	1833	+	ggccctcccct
MA0079.3	SP1	11.5693	0.926696160379	124	134	+	gctcctccctt
